# Supplementary figures and images for: Phosphate starvation signaling increases mitochondrial membrane potential through respiration-independent mechanisms
Source: eLife. 2024 Jan 22;13:e84282. doi: 10.7554/eLife.84282 (PMC10846858; doi:10.7554/eLife.84282)

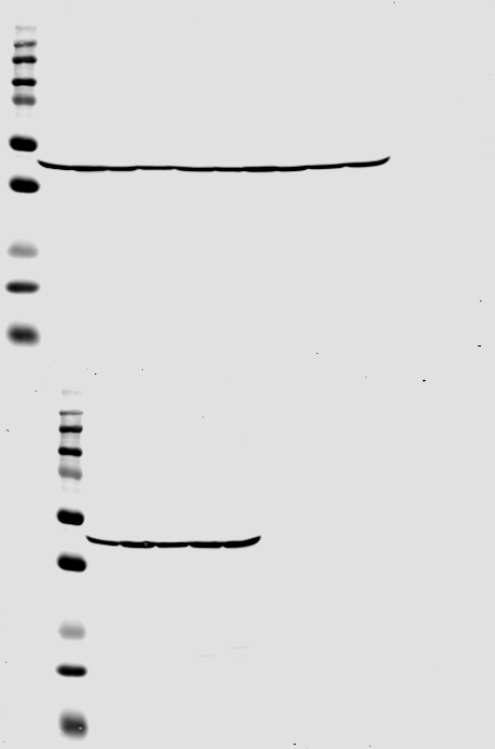

Supplement: Figure 1—source data 1. [file elife-84282-fig1-data1.zip › Figure 1--source data/raw data file4_Fig1_pgk1_ilv2-flag_pho85mct1_sit4mct1_triplicate_7.19.22.png]

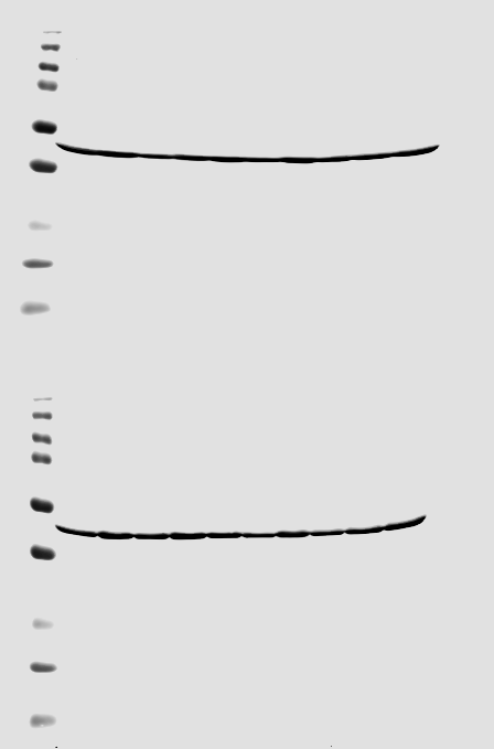

Supplement: Figure 1—source data 1. [file elife-84282-fig1-data1.zip › Figure 1--source data/raw data file2_Fig1_Pgk1_ilv2-flag_sit4mct1_triplicate_7.12.22.png]

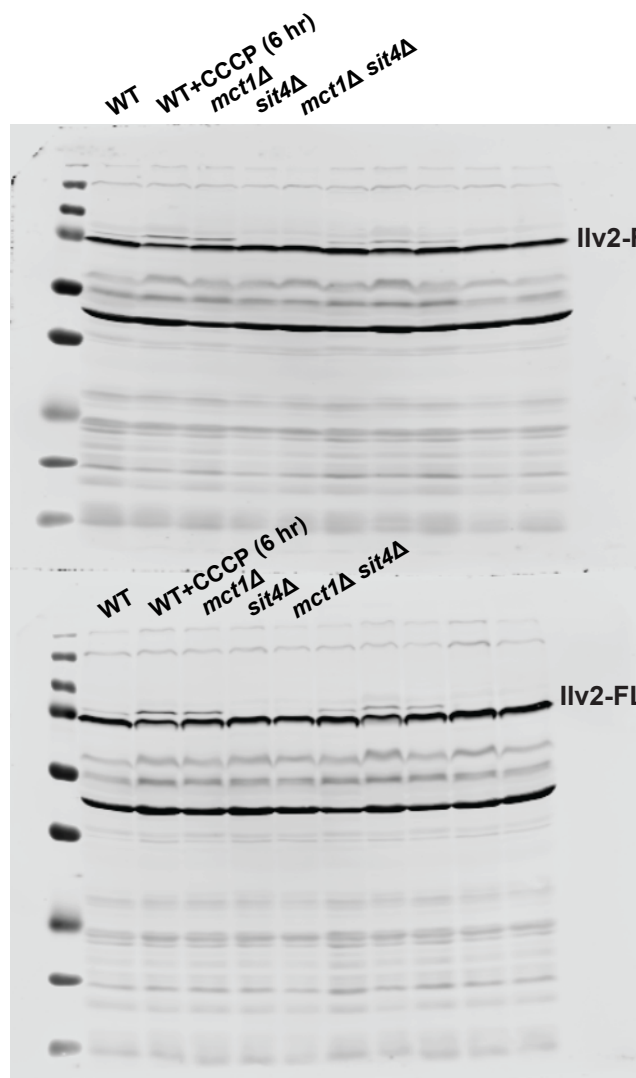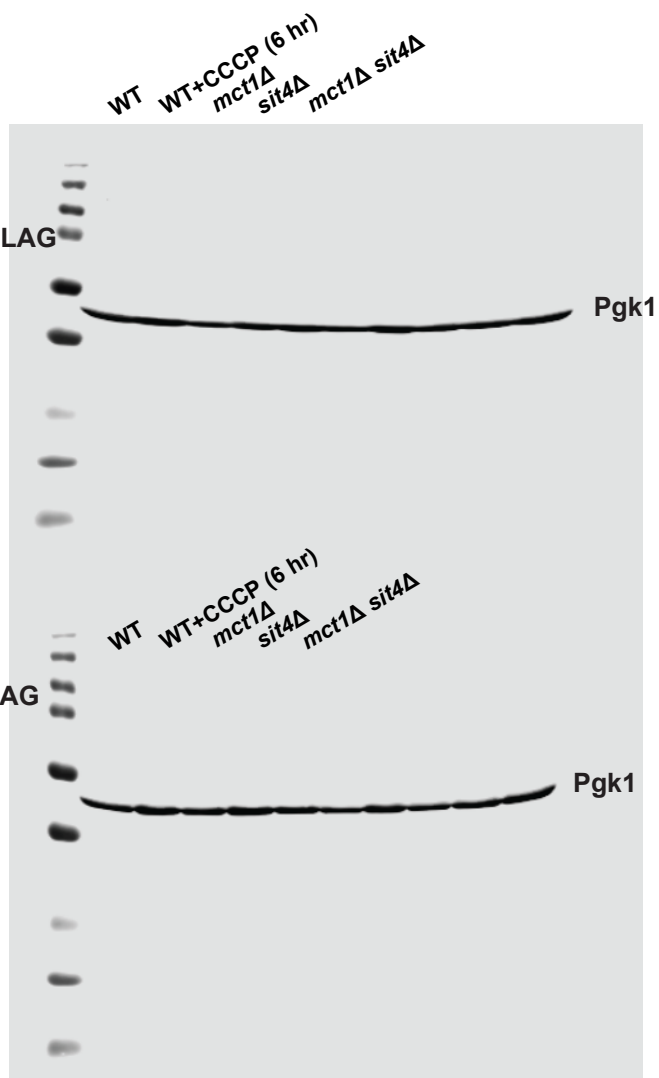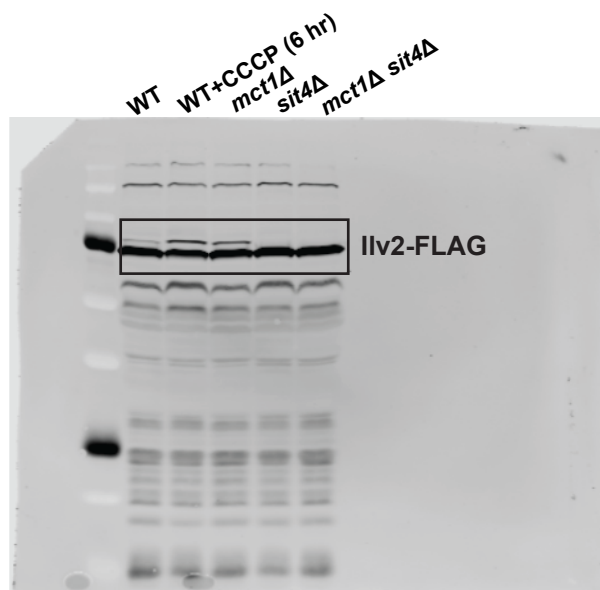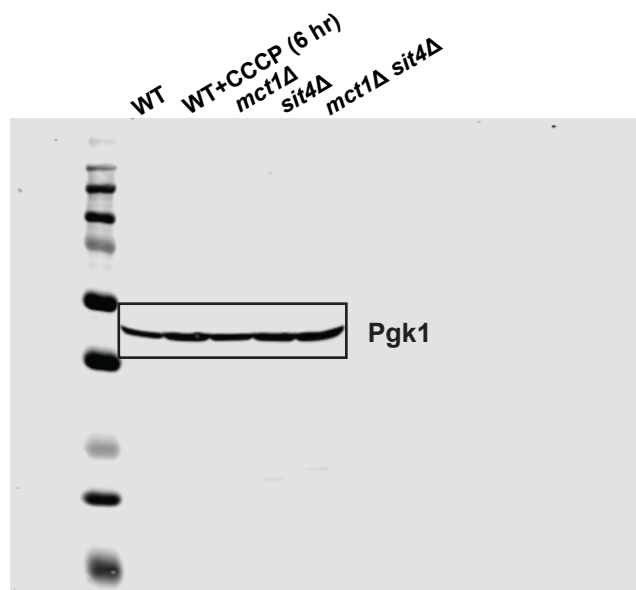

Supplement: Figure 1—source data 1. [file elife-84282-fig1-data1.zip › Figure 1--source data/Figure1--source data.pdf]

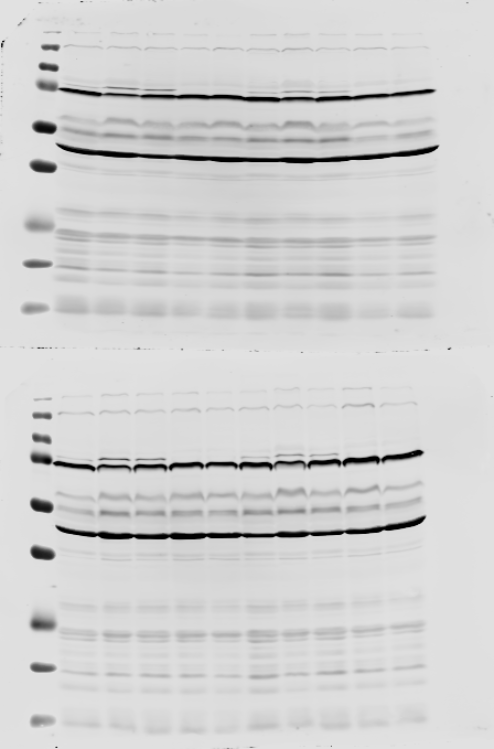

Supplement: Figure 1—source data 1. [file elife-84282-fig1-data1.zip › Figure 1--source data/raw data file1_Fig1_ilv2-flag_sit4mct1_triplicate_7.12.22.png]

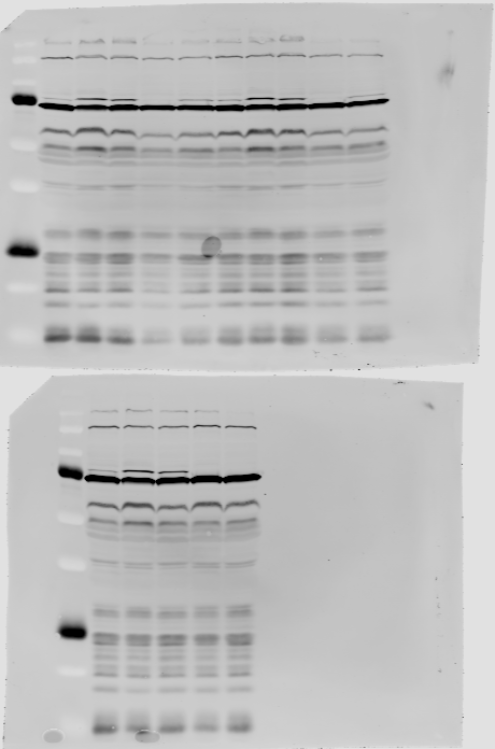

Supplement: Figure 1—source data 1. [file elife-84282-fig1-data1.zip › Figure 1--source data/raw data file3_Fig1_ilv2-flag_pho85mct1_sit4mct1_triplicate_7.19.22.png]

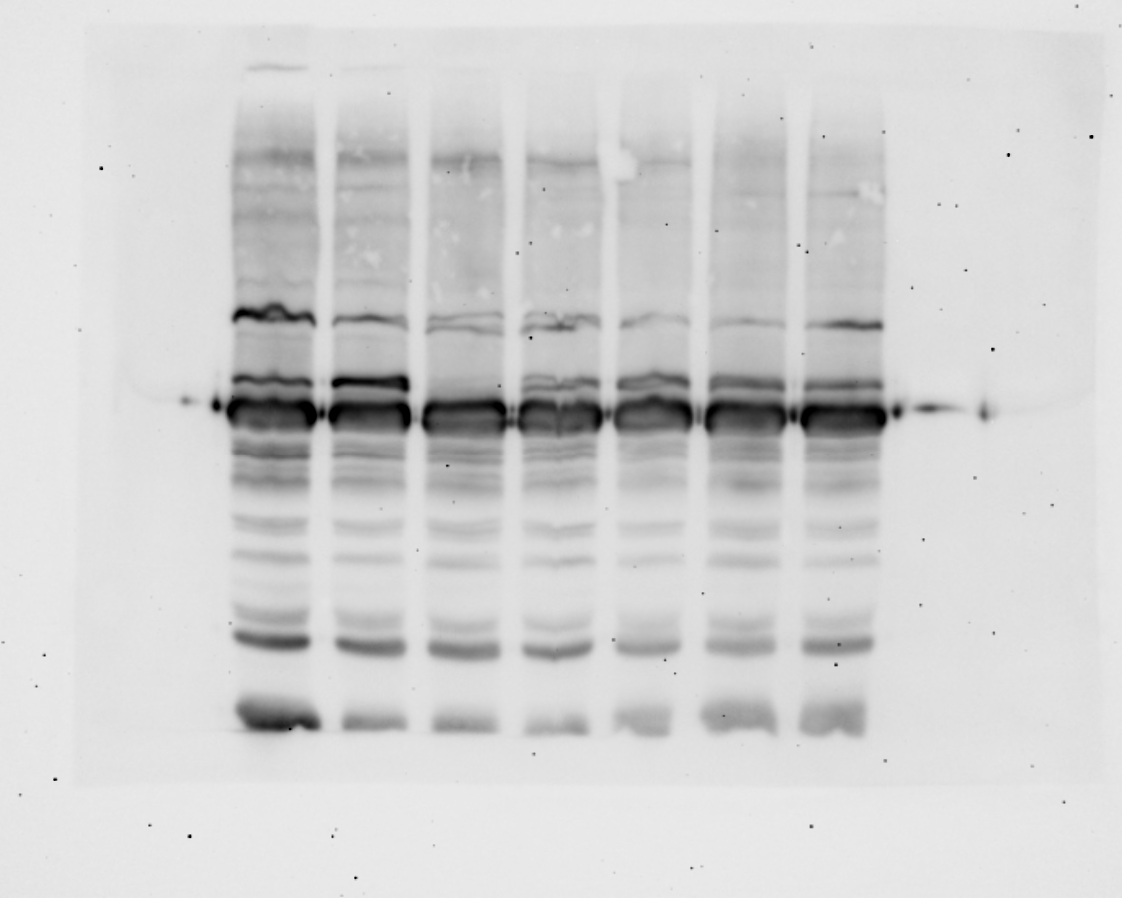

Supplement: Figure 1—figure supplement 1—source data 1. [file elife-84282-fig1-figsupp1-data1.zip › Figure 1-figure supplement 1-source data 1/raw data file1_FigS1_2023-07-07 190611 BNP-3(Chemiluminescence).tif]

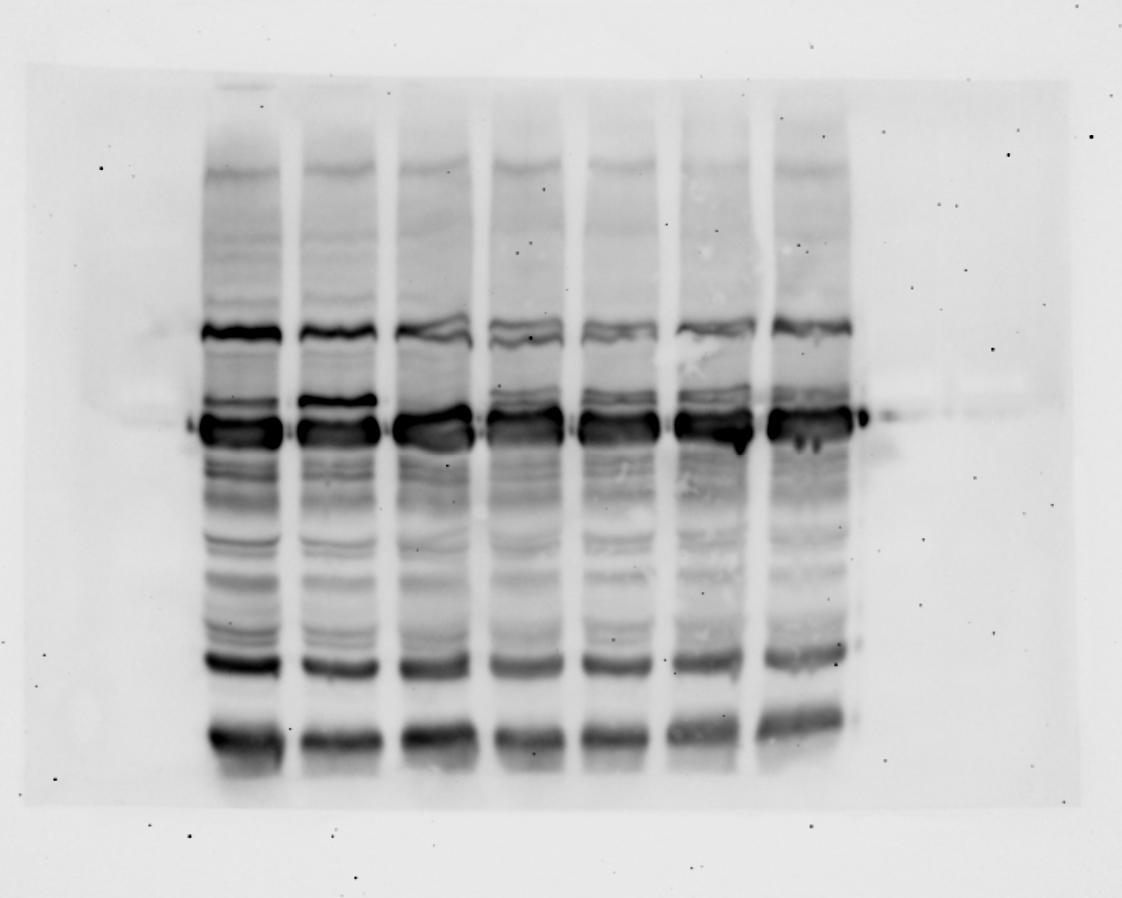

Supplement: Figure 1—figure supplement 1—source data 1. [file elife-84282-fig1-figsupp1-data1.zip › Figure 1-figure supplement 1-source data 1/raw data file3_FigS1_2023-07-07 190611 BNP-3(Chemiluminescence).tif]

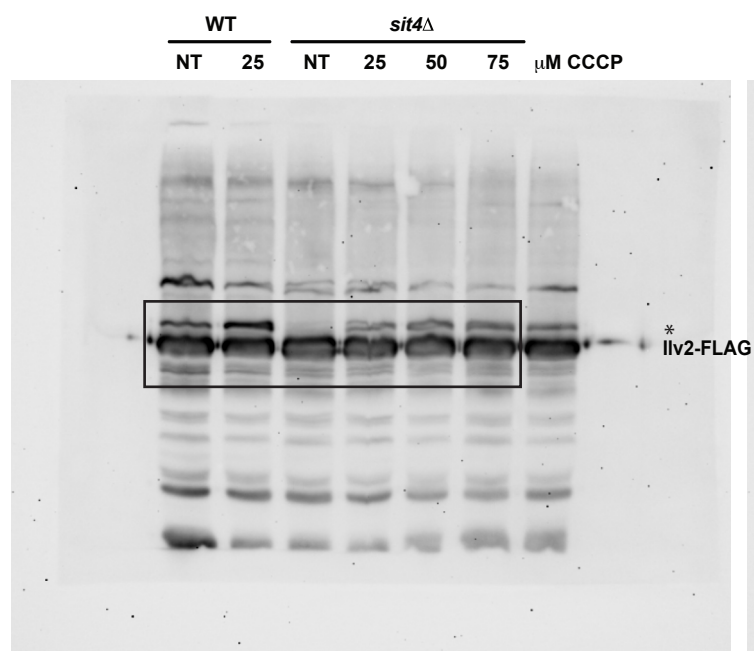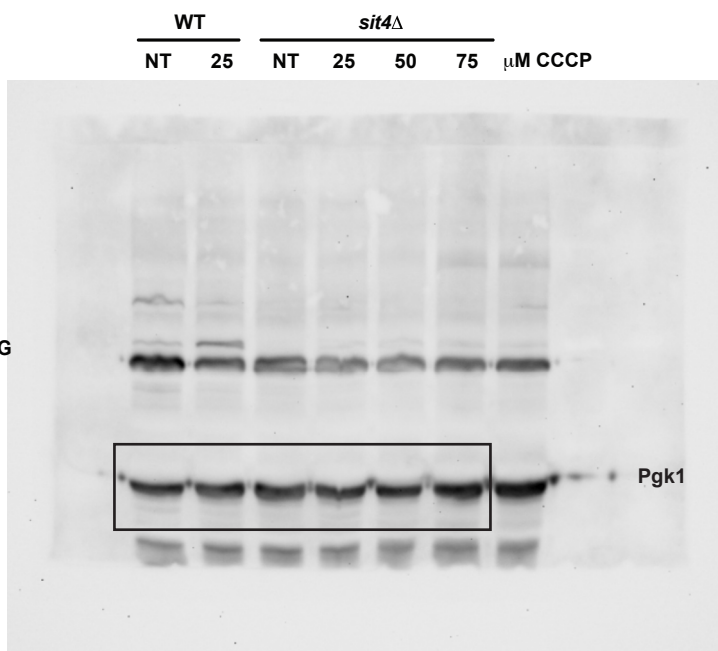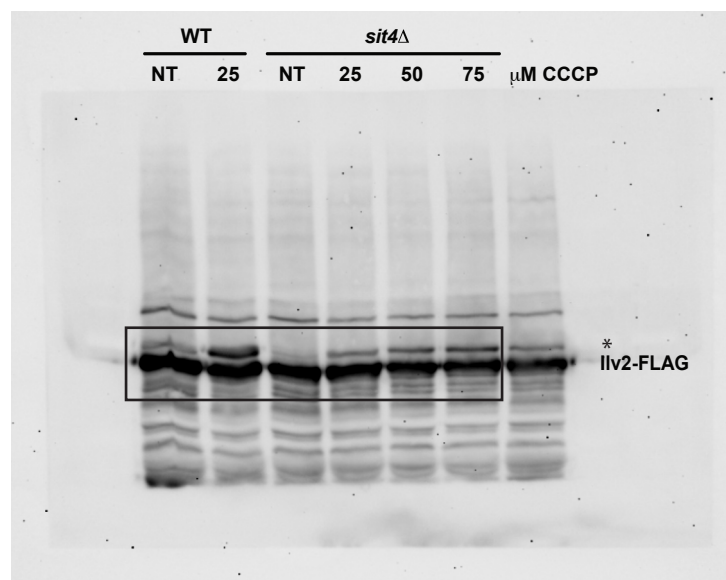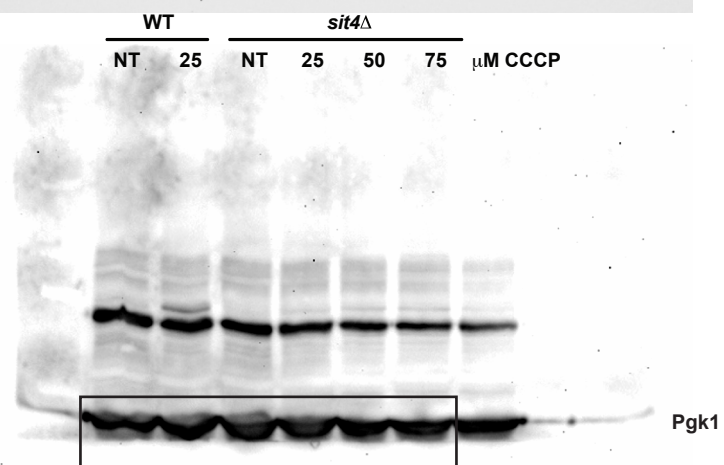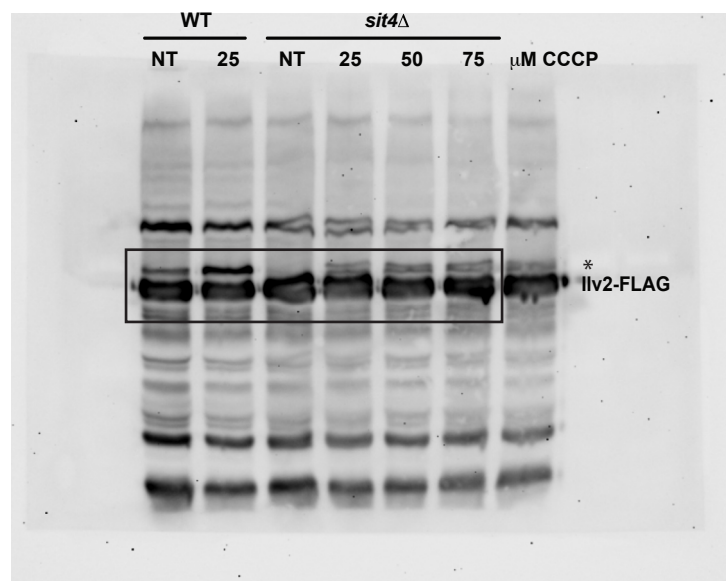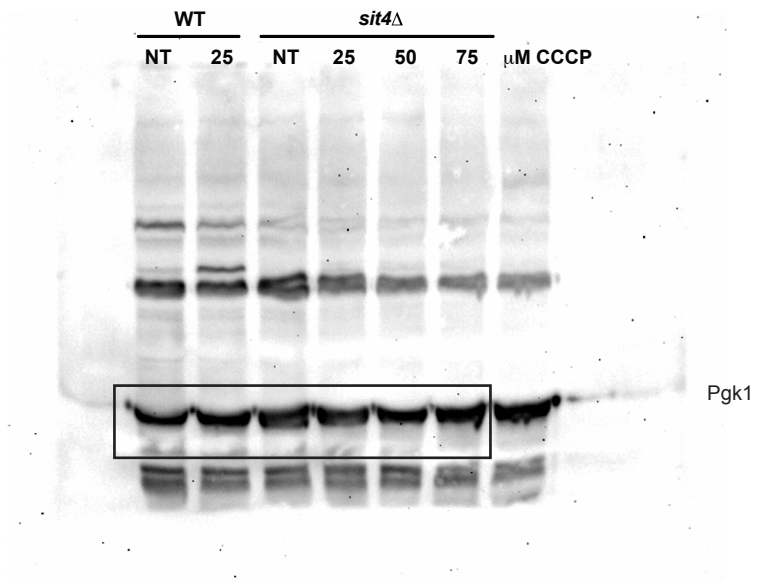

Supplement: Figure 1—figure supplement 1—source data 1. [file elife-84282-fig1-figsupp1-data1.zip › Figure 1-figure supplement 1-source data 1/raw data associated with Figure 1-figure supplement 1.pdf]

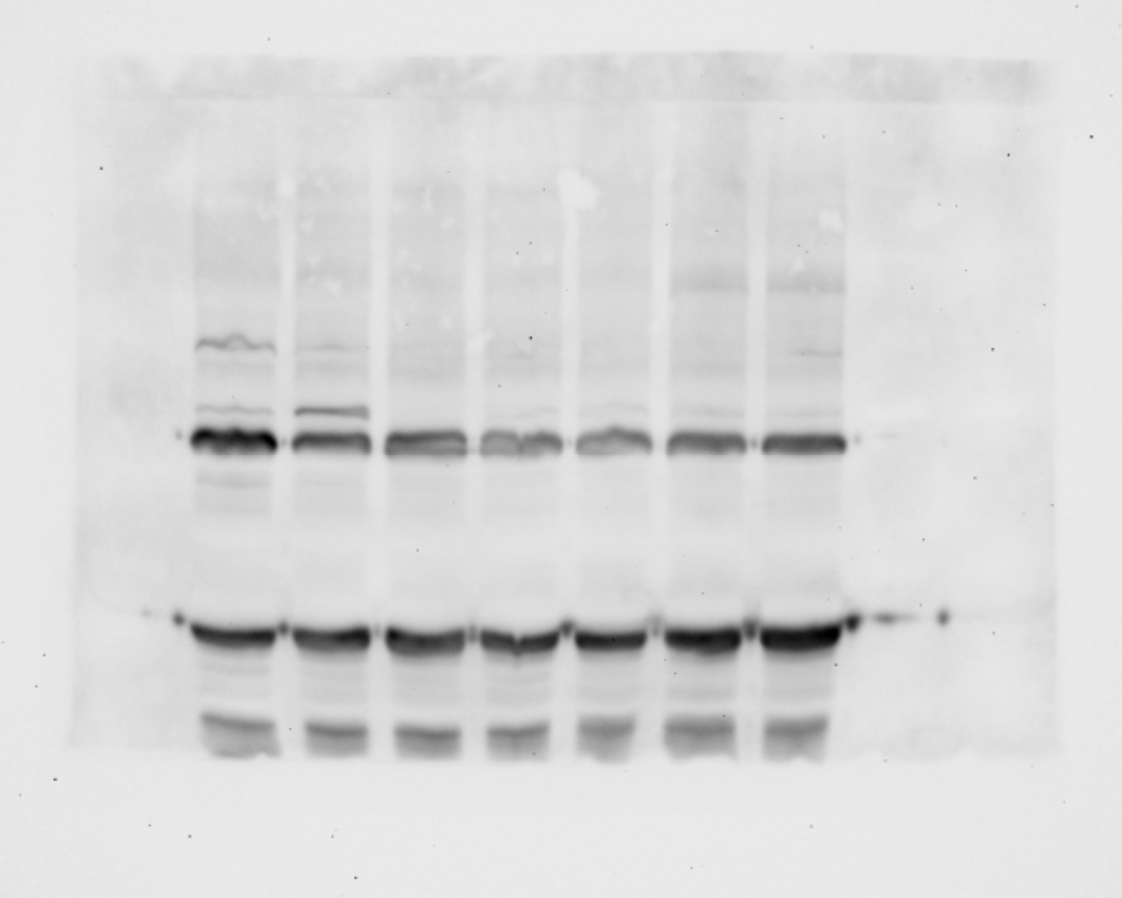

Supplement: Figure 1—figure supplement 1—source data 1. [file elife-84282-fig1-figsupp1-data1.zip › Figure 1-figure supplement 1-source data 1/raw data file4_FigS1_2023-07-10 190611 Blot1 pgk1(Chemiluminescence).tif]

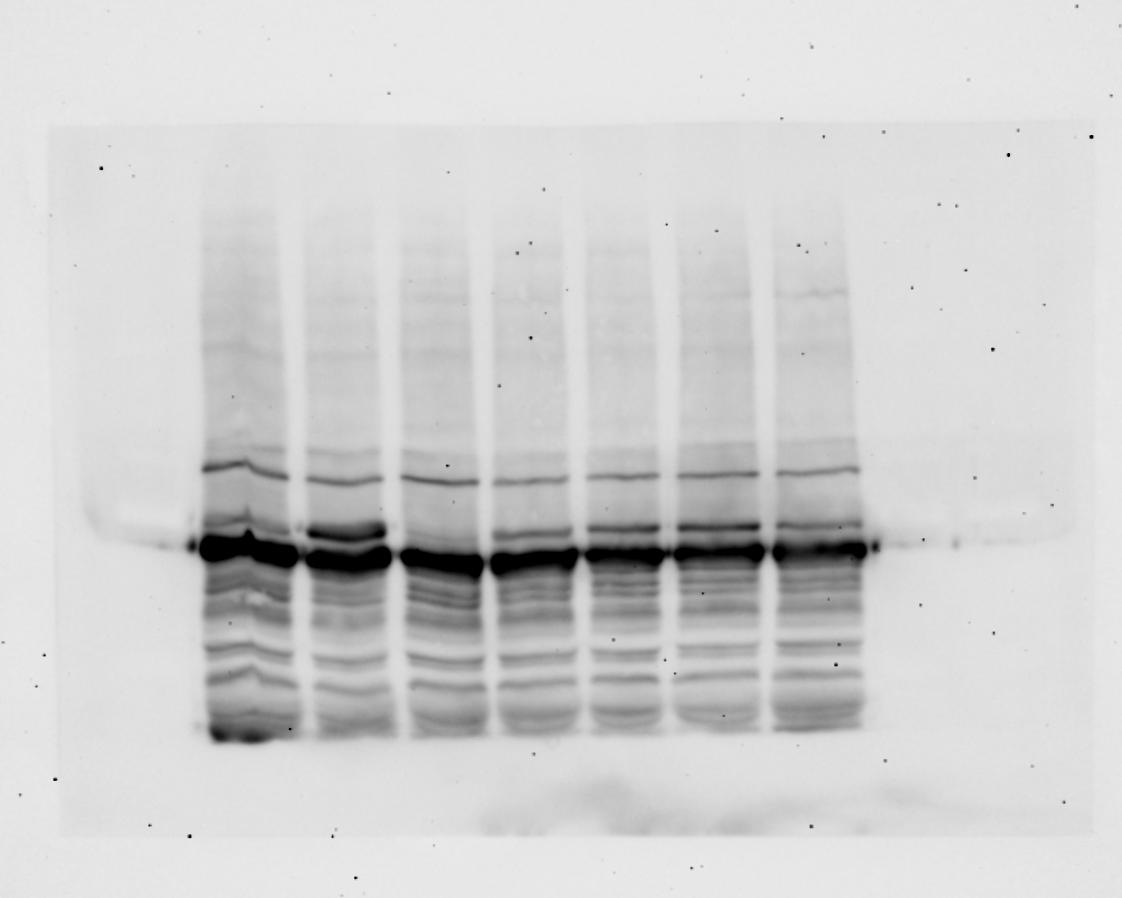

Supplement: Figure 1—figure supplement 1—source data 1. [file elife-84282-fig1-figsupp1-data1.zip › Figure 1-figure supplement 1-source data 1/raw data file2_FigS1_2023-07-07 190611 BNP-4(Chemiluminescence).tif]

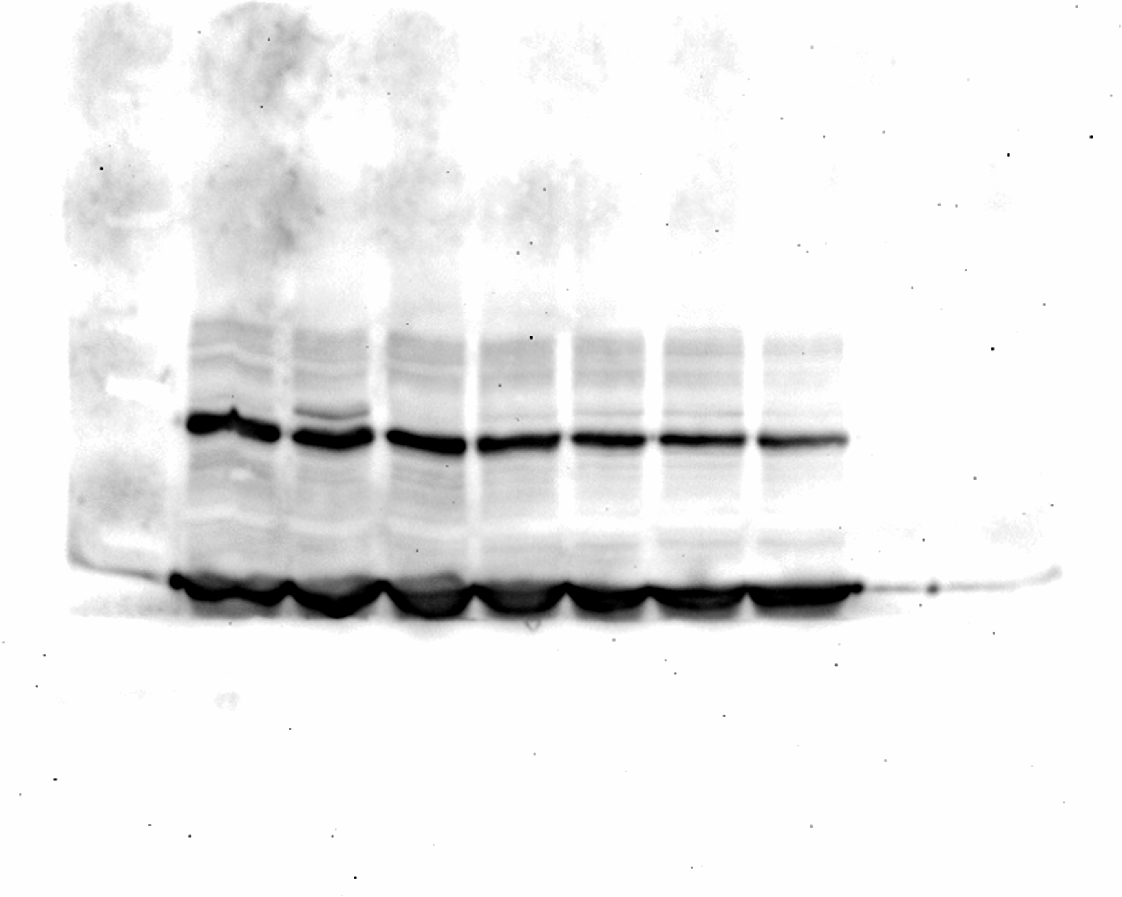

Supplement: Figure 1—figure supplement 1—source data 1. [file elife-84282-fig1-figsupp1-data1.zip › Figure 1-figure supplement 1-source data 1/raw data file5_FigS1_2023-07-10 190611 Blot2 pgk1(Chemiluminescence).tif]

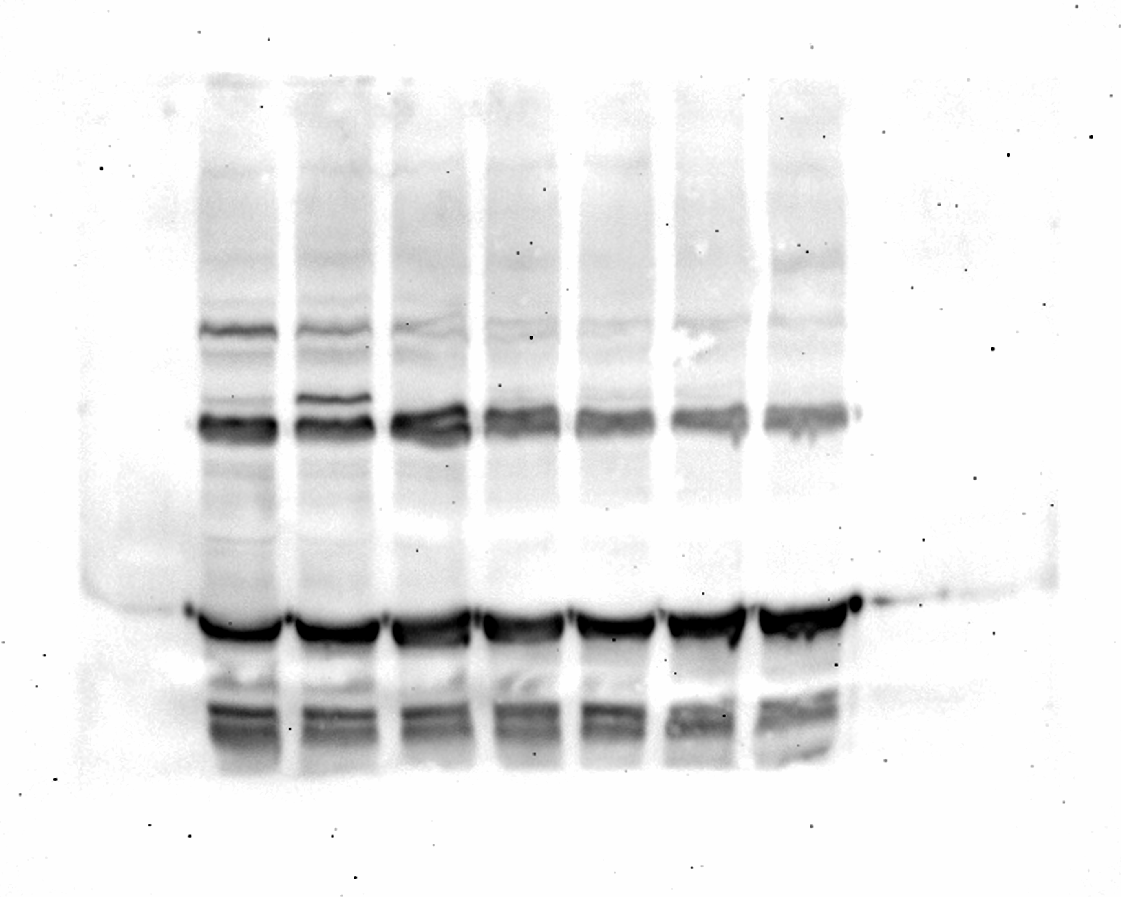

Supplement: Figure 1—figure supplement 1—source data 1. [file elife-84282-fig1-figsupp1-data1.zip › Figure 1-figure supplement 1-source data 1/raw data file6_FigS1_2023-07-10 190611 blot3 pgk1(Chemiluminescence).tif]

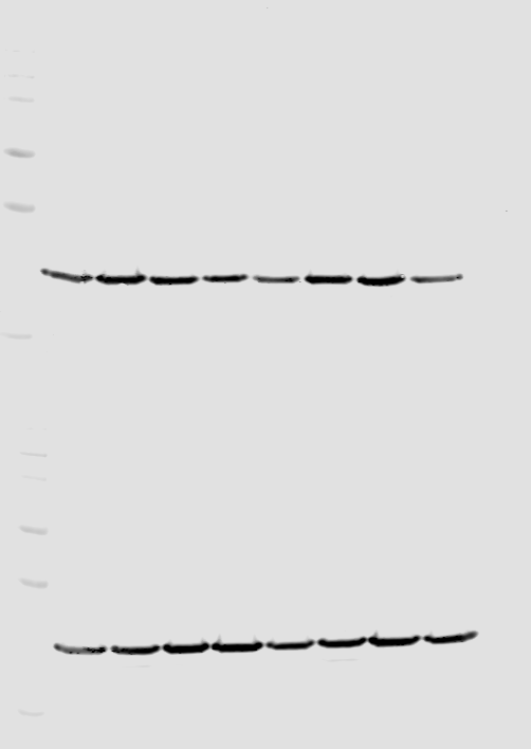

Supplement: Figure 2—source data 1. [file elife-84282-fig2-data1.zip › Figure 2-source data 1/raw data file3_Fig2_por1_ctrl_Pi_BNPAGE_7.24.22.png]

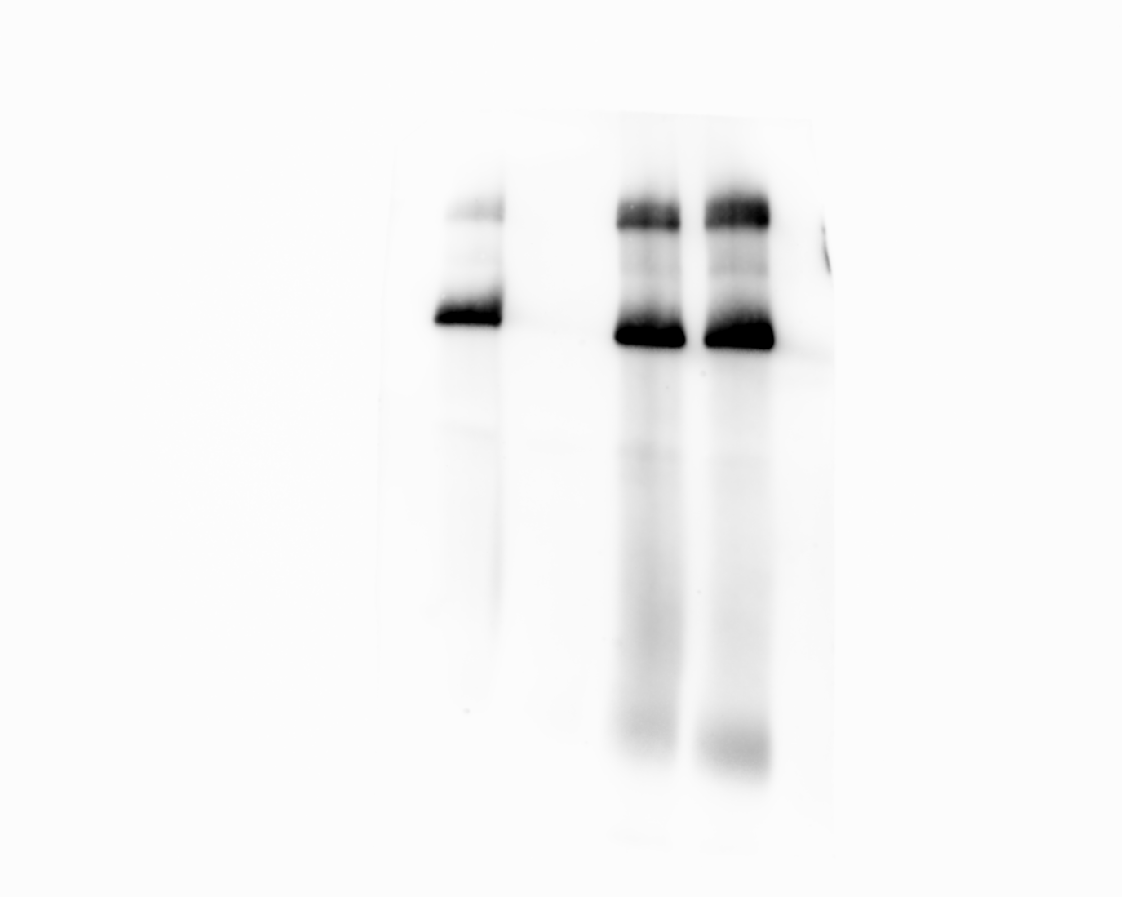

Supplement: Figure 2—source data 1. [file elife-84282-fig2-data1.zip › Figure 2-source data 1/raw data file1_Fig2_2022-07-22 190611 BNP-1(Chemiluminescence).tif]

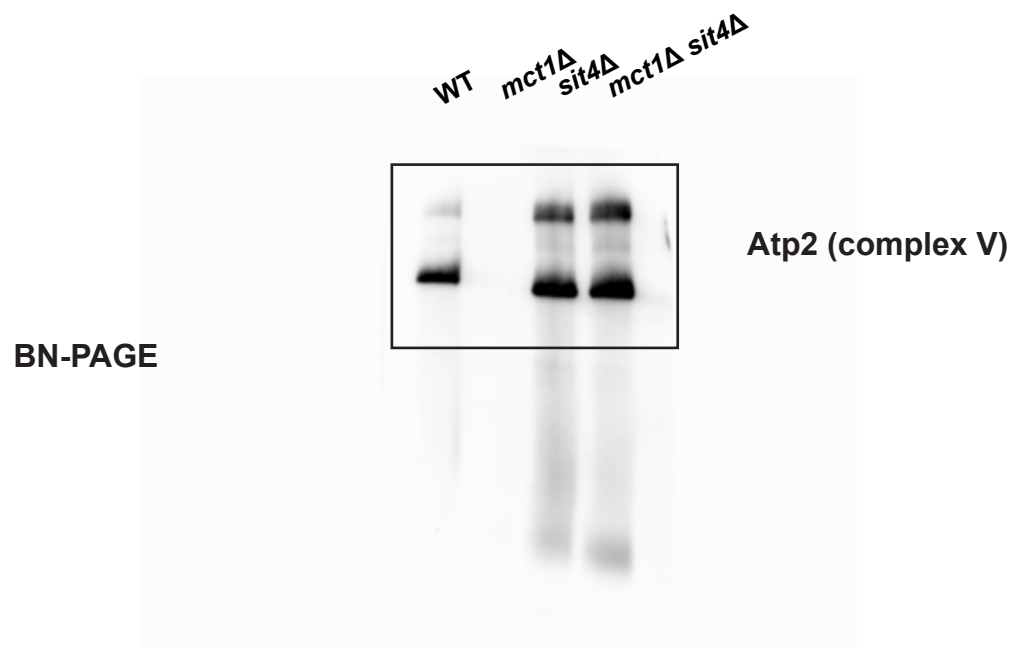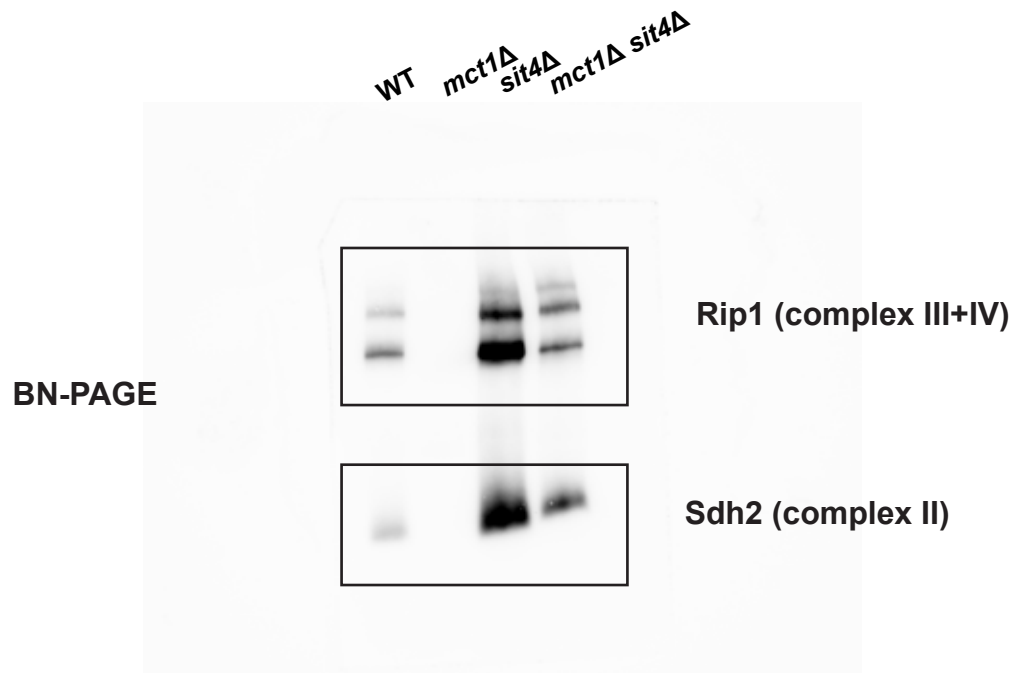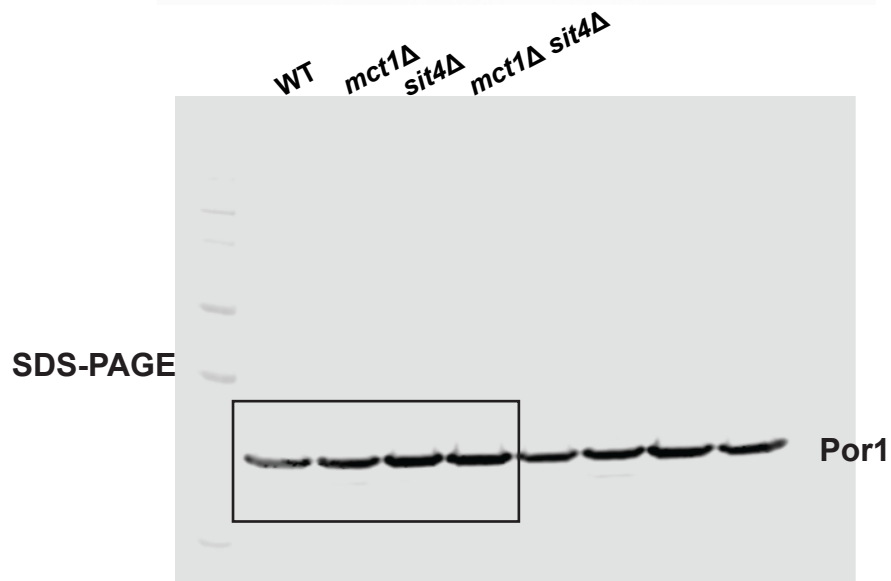

Supplement: Figure 2—source data 1. [file elife-84282-fig2-data1.zip › Figure 2-source data 1/raw data associated with Figure 2.pdf]

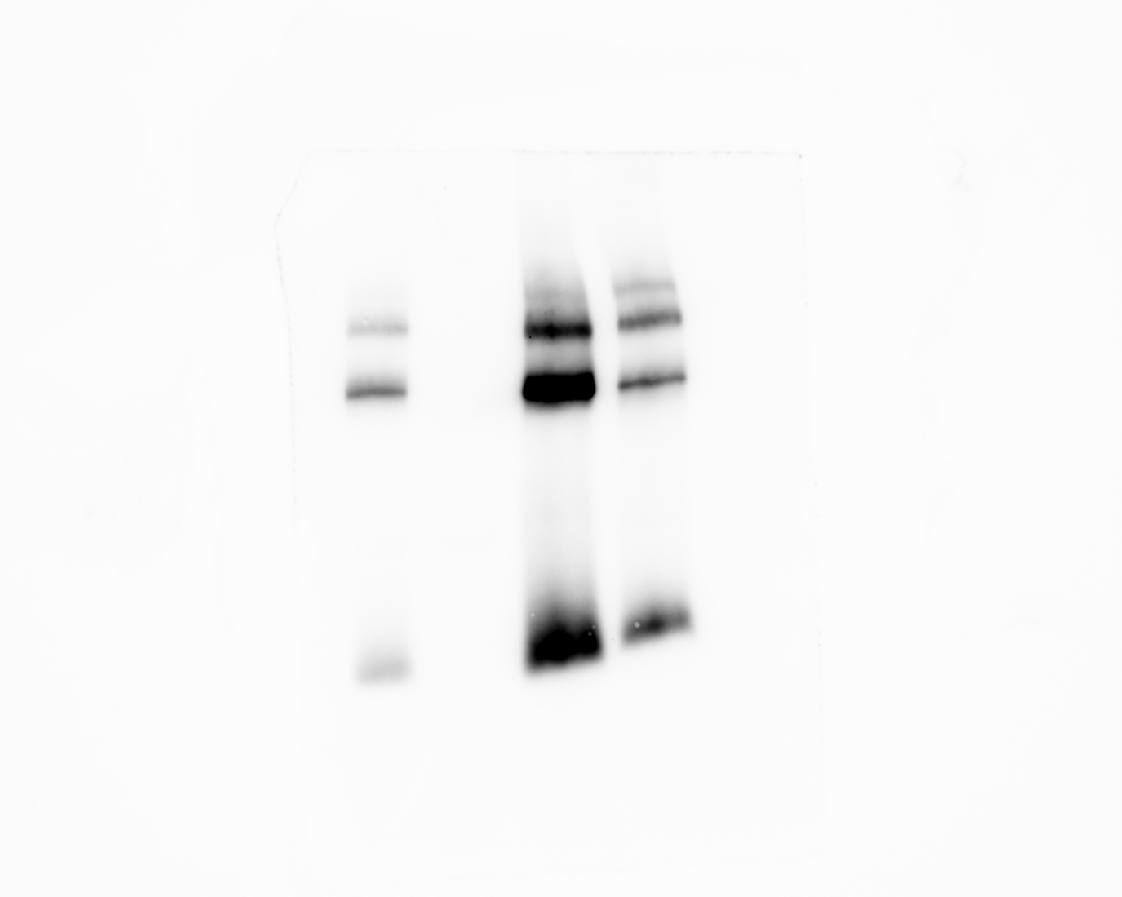

Supplement: Figure 2—source data 1. [file elife-84282-fig2-data1.zip › Figure 2-source data 1/raw data file2_Fig2_2022-07-23 190611 BNP-4(Chemiluminescence).tif]

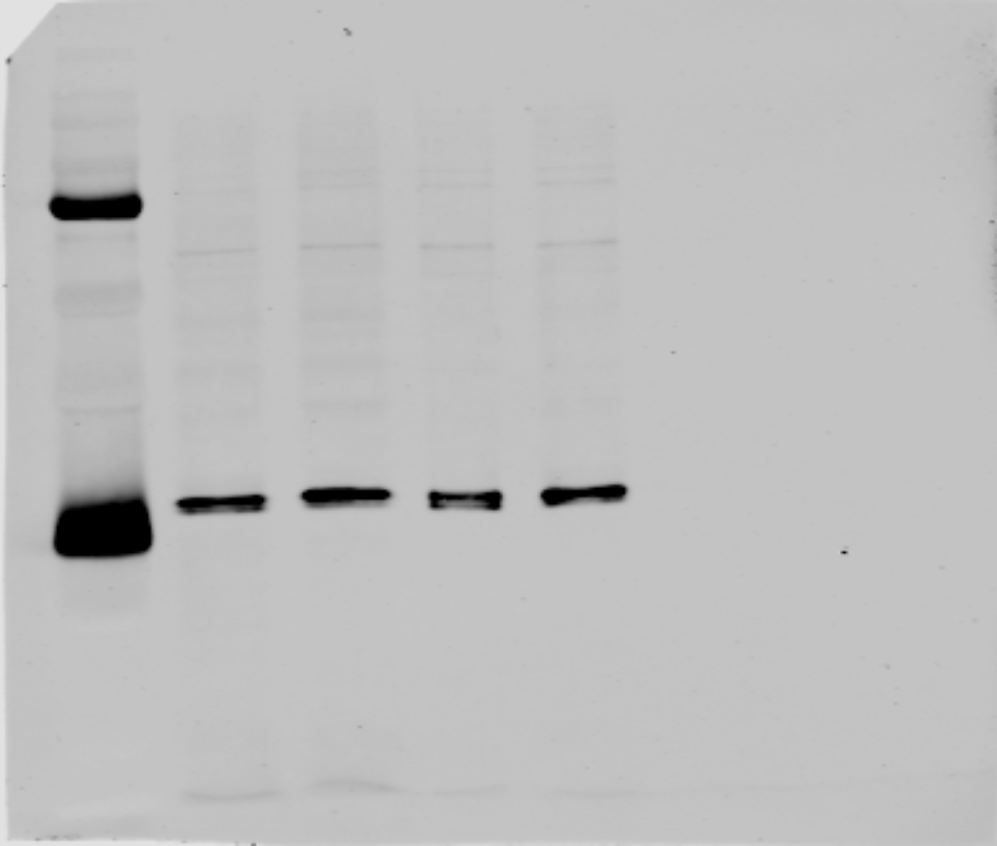

Supplement: Figure 2—figure supplement 1—source data 1. [file elife-84282-fig2-figsupp1-data1.zip › Figure 2-figure supplement 1-source data 1/raw data file12_FigS2_por1_ctrl_Pi_BNPAGE_10.21.22.png]

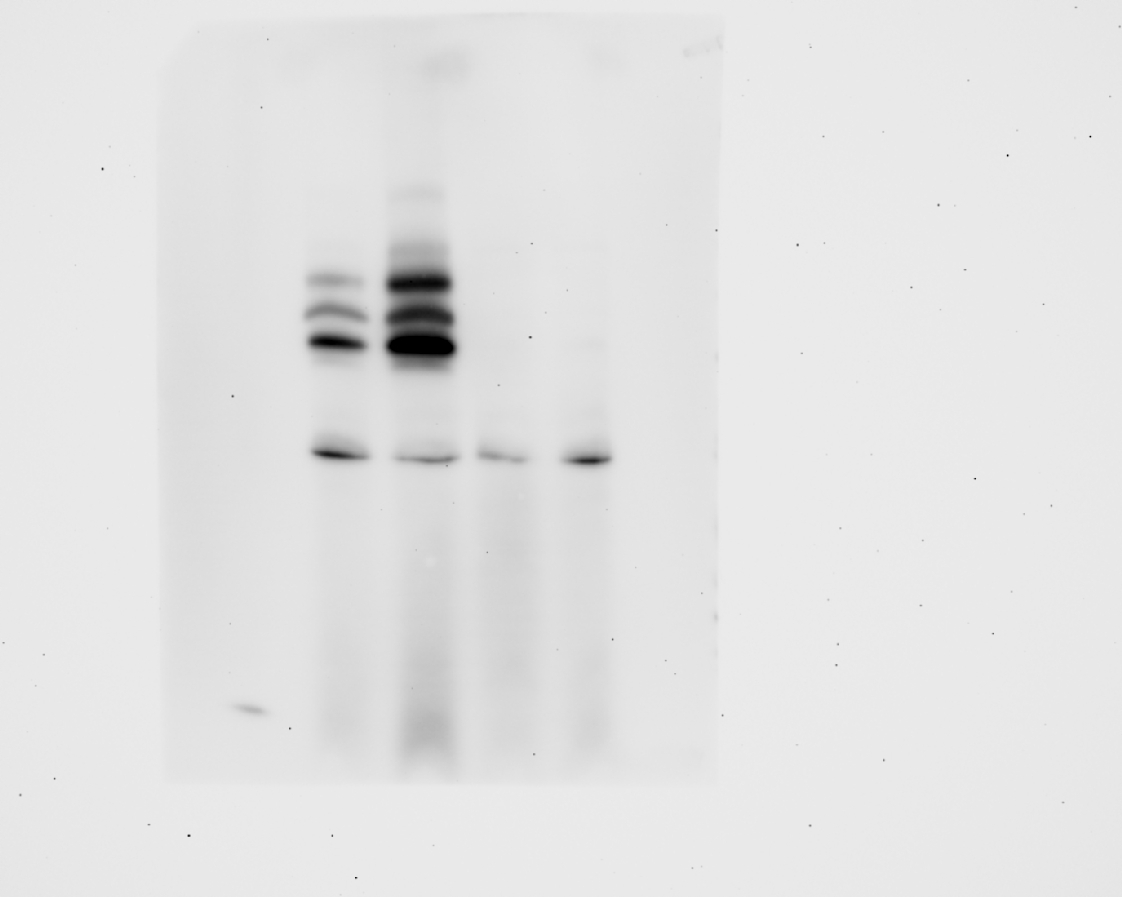

Supplement: Figure 2—figure supplement 1—source data 1. [file elife-84282-fig2-figsupp1-data1.zip › Figure 2-figure supplement 1-source data 1/raw data file8_FigS2Hsp60_HAP4OE_BN-PAGE_4.27.23.jpg]

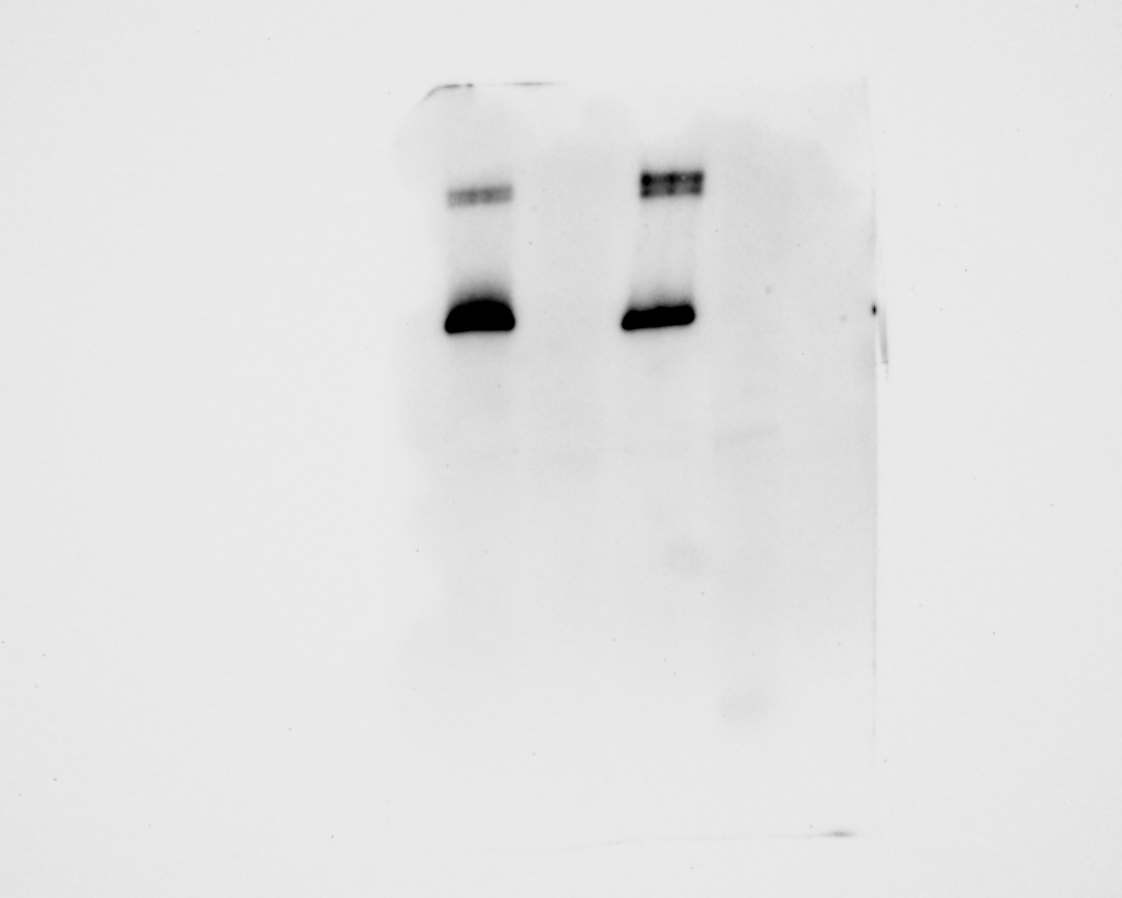

Supplement: Figure 2—figure supplement 1—source data 1. [file elife-84282-fig2-figsupp1-data1.zip › Figure 2-figure supplement 1-source data 1/raw data file10_FigS2_2022-10-21 190611 BNP-5(Chemiluminescence).tif]

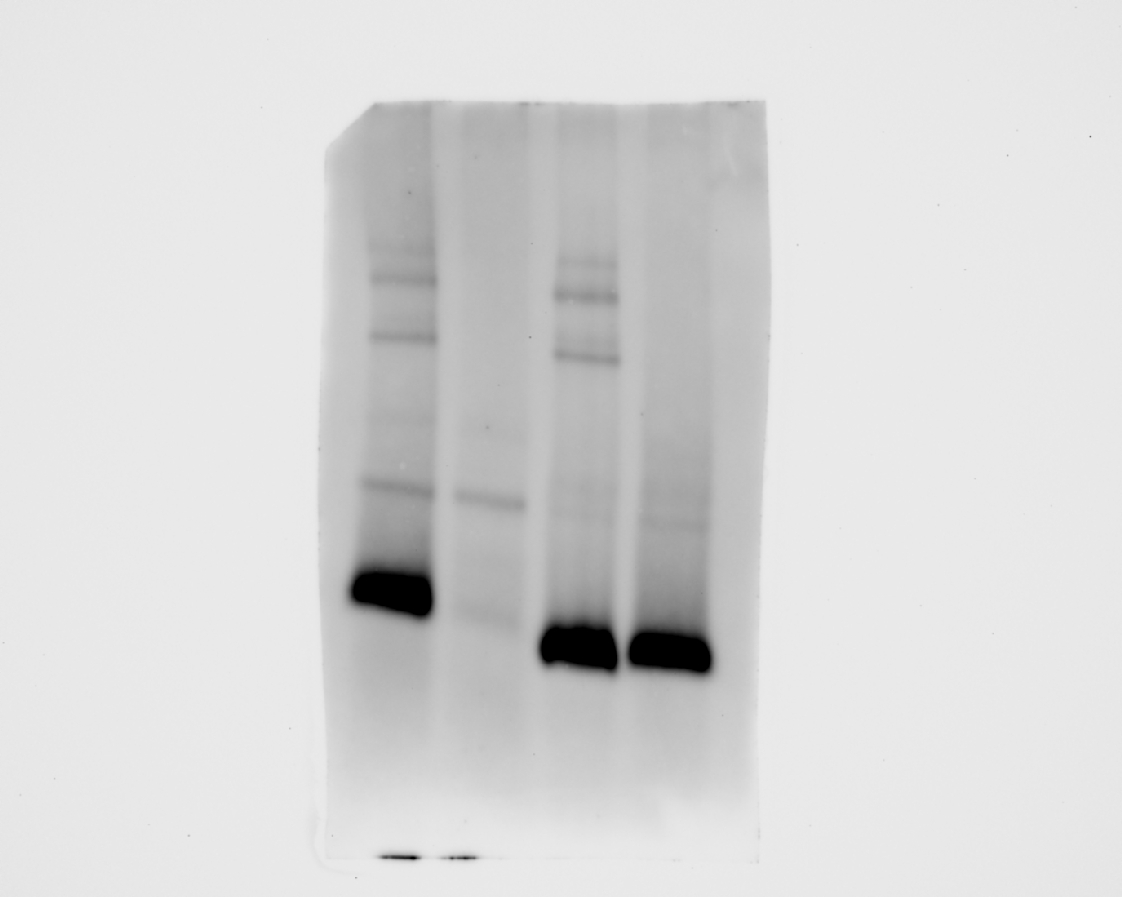

Supplement: Figure 2—figure supplement 1—source data 1. [file elife-84282-fig2-figsupp1-data1.zip › Figure 2-figure supplement 1-source data 1/raw data file11_FigS2_2022-10-23 190611 BNP-2(Chemiluminescence).jpg]

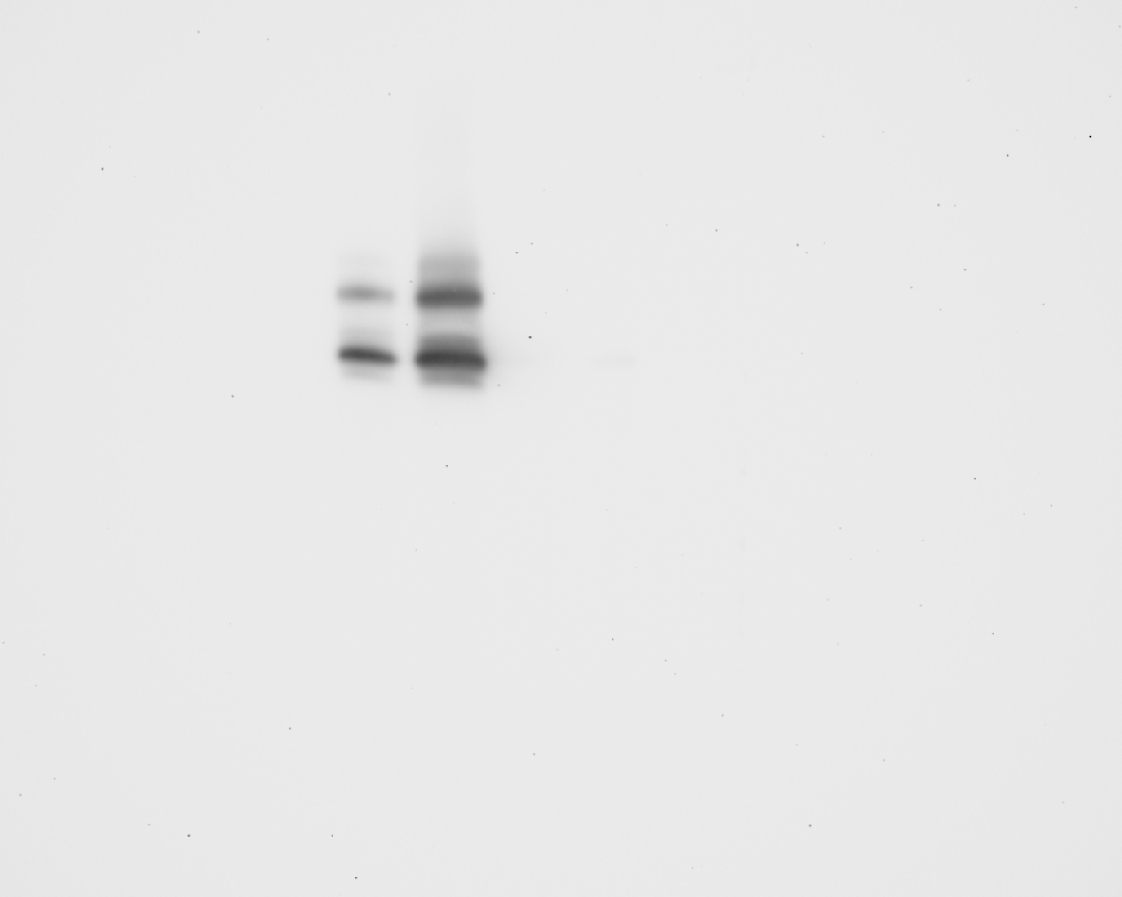

Supplement: Figure 2—figure supplement 1—source data 1. [file elife-84282-fig2-figsupp1-data1.zip › Figure 2-figure supplement 1-source data 1/raw data file6_FigS2Rip1_HAP4OE_BN-PAGE_4.27.23.jpg]

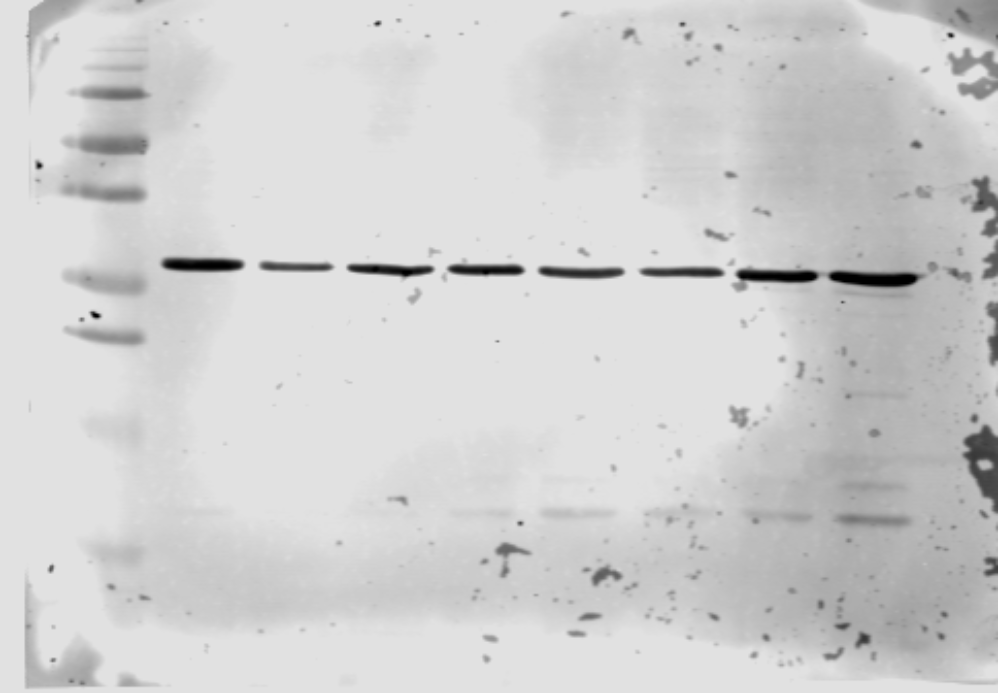

Supplement: Figure 2—figure supplement 1—source data 1. [file elife-84282-fig2-figsupp1-data1.zip › Figure 2-figure supplement 1-source data 1/raw data file2_FigS2_por1_acyl-acp_sit4mct1_1.23.20.png]

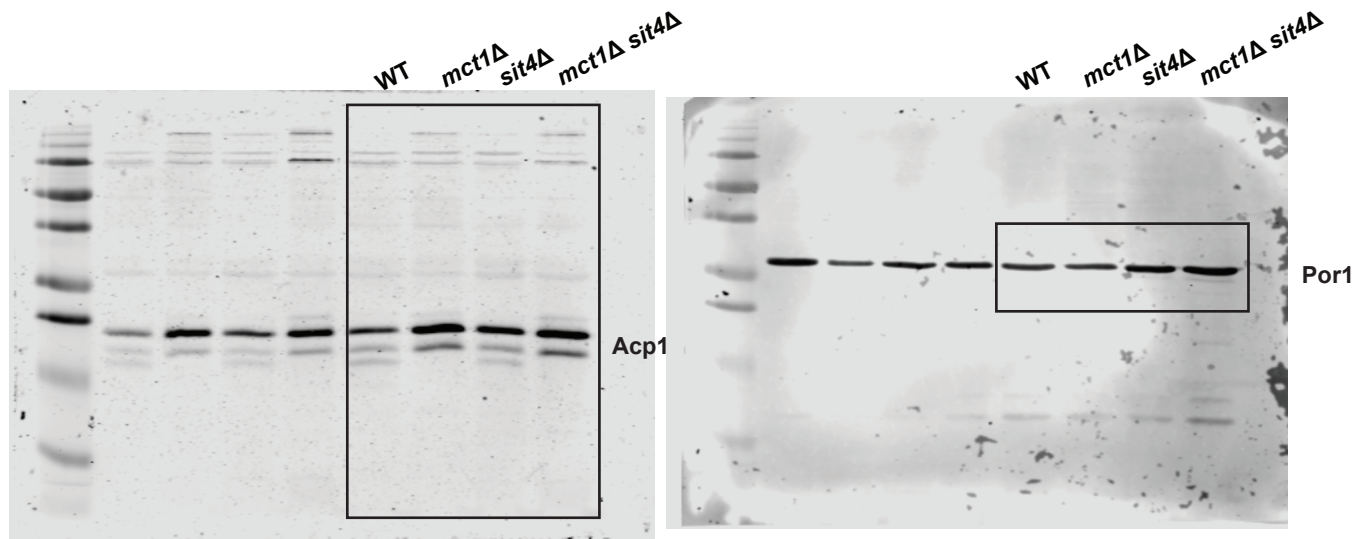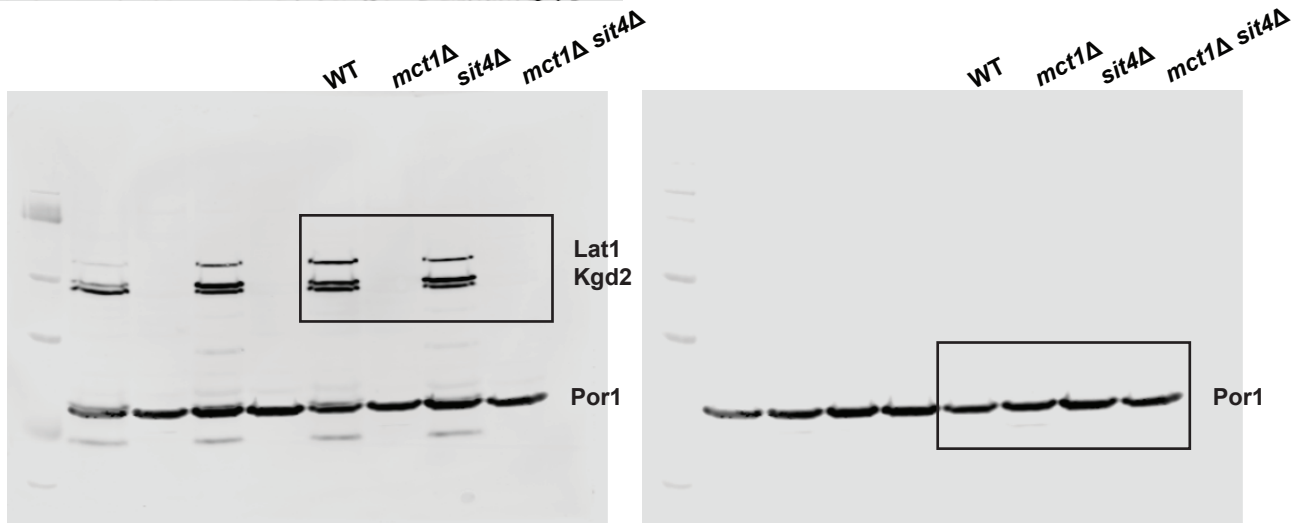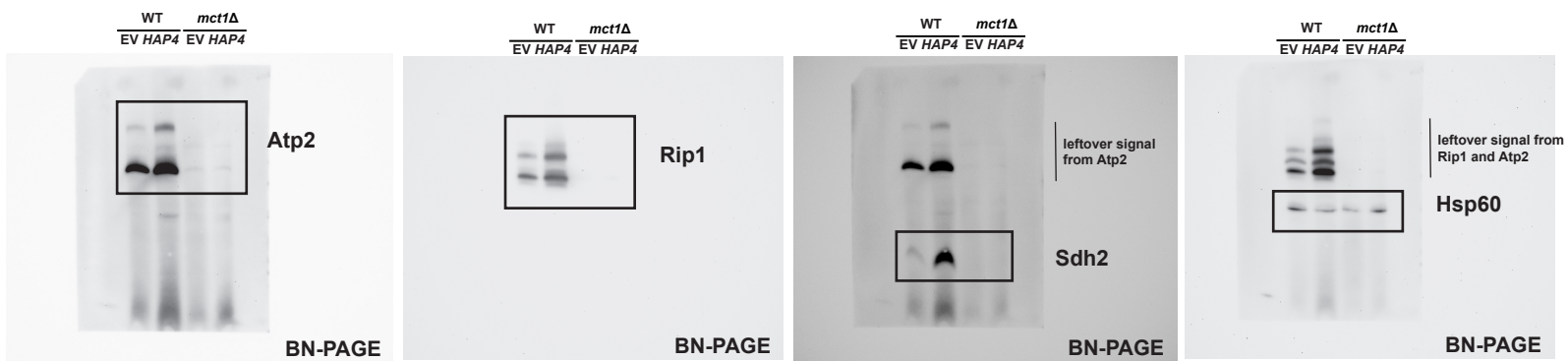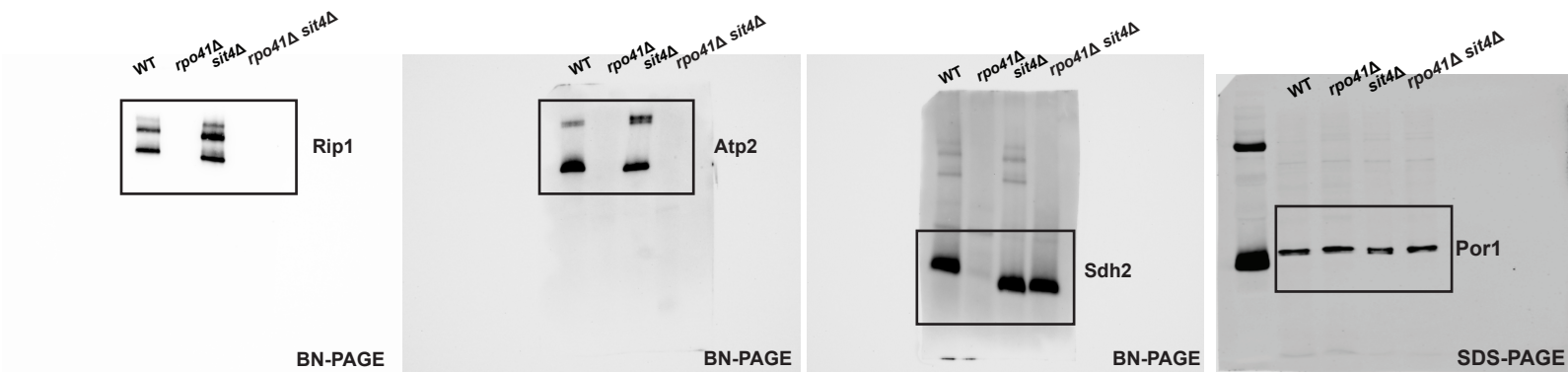

Supplement: Figure 2—figure supplement 1—source data 1. [file elife-84282-fig2-figsupp1-data1.zip › Figure 2-figure supplement 1-source data 1/raw data associated with Figure 2-figure supplement 1.pdf]

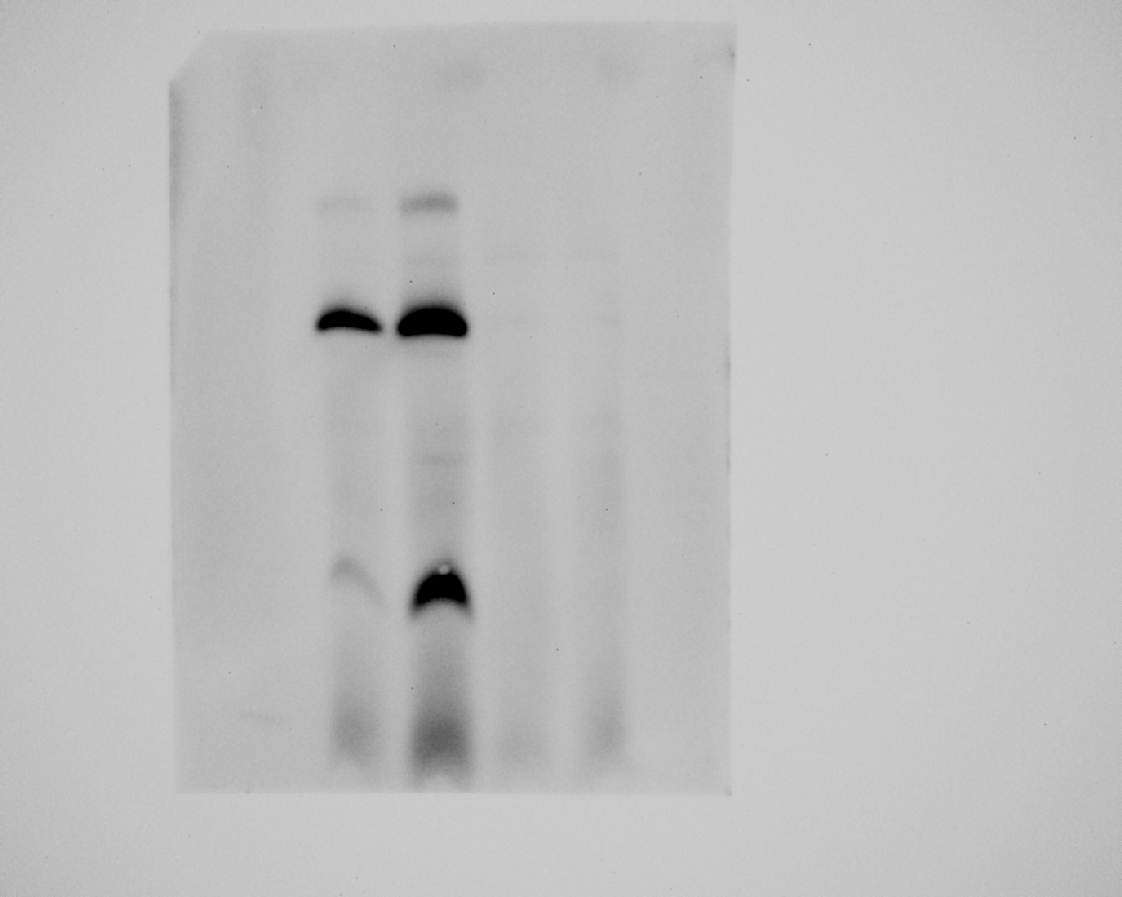

Supplement: Figure 2—figure supplement 1—source data 1. [file elife-84282-fig2-figsupp1-data1.zip › Figure 2-figure supplement 1-source data 1/raw data file7_sdh2_HAP4OE_BN-PAGE_4.27.23.jpg]

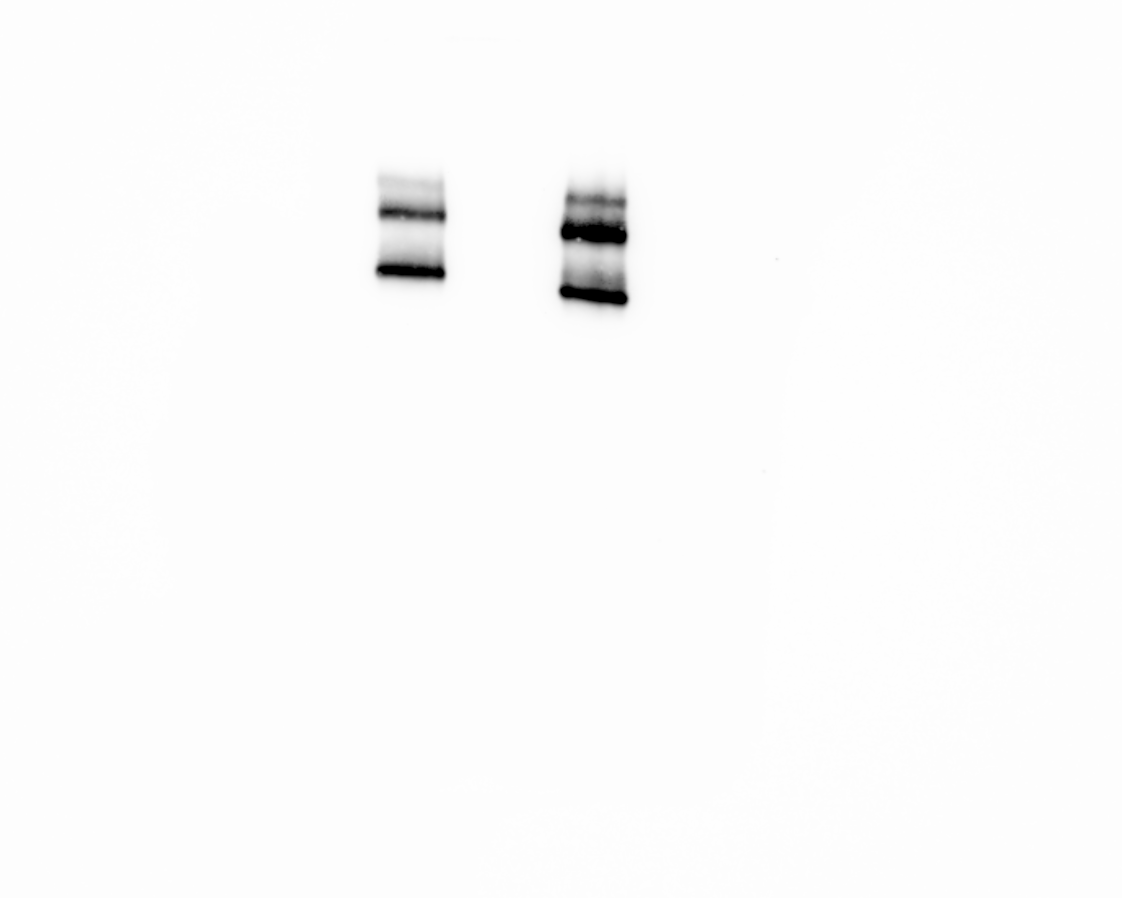

Supplement: Figure 2—figure supplement 1—source data 1. [file elife-84282-fig2-figsupp1-data1.zip › Figure 2-figure supplement 1-source data 1/raw data file9_Figs2_2022-10-21 190611 BNP-3(Chemiluminescence).tif]

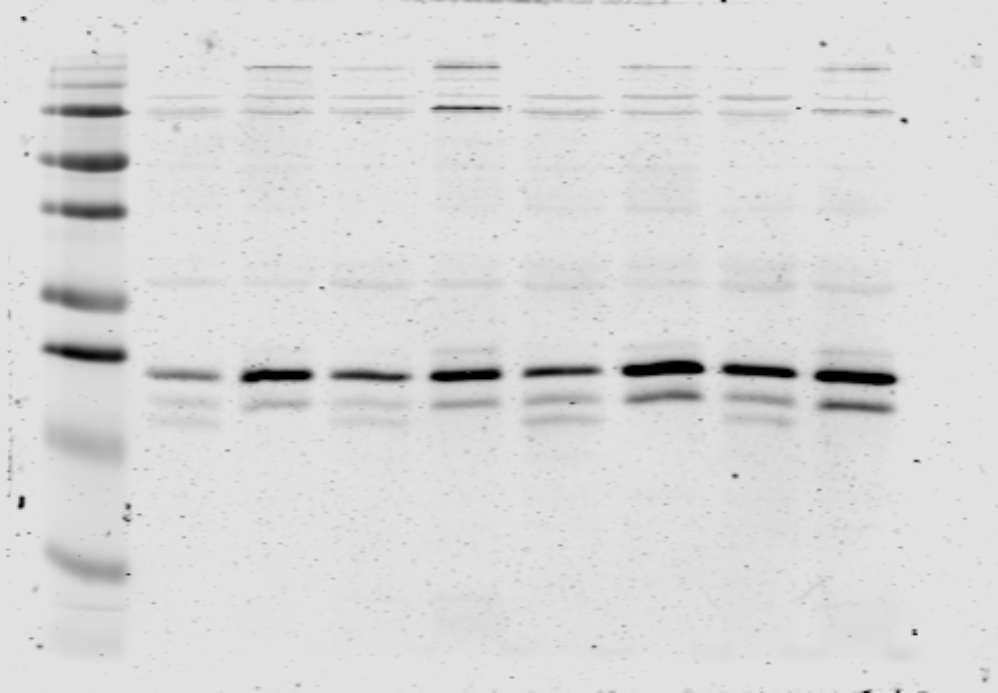

Supplement: Figure 2—figure supplement 1—source data 1. [file elife-84282-fig2-figsupp1-data1.zip › Figure 2-figure supplement 1-source data 1/raw data file1_FigS2_acyl-acp_sit4mct1_1.23.20.png]

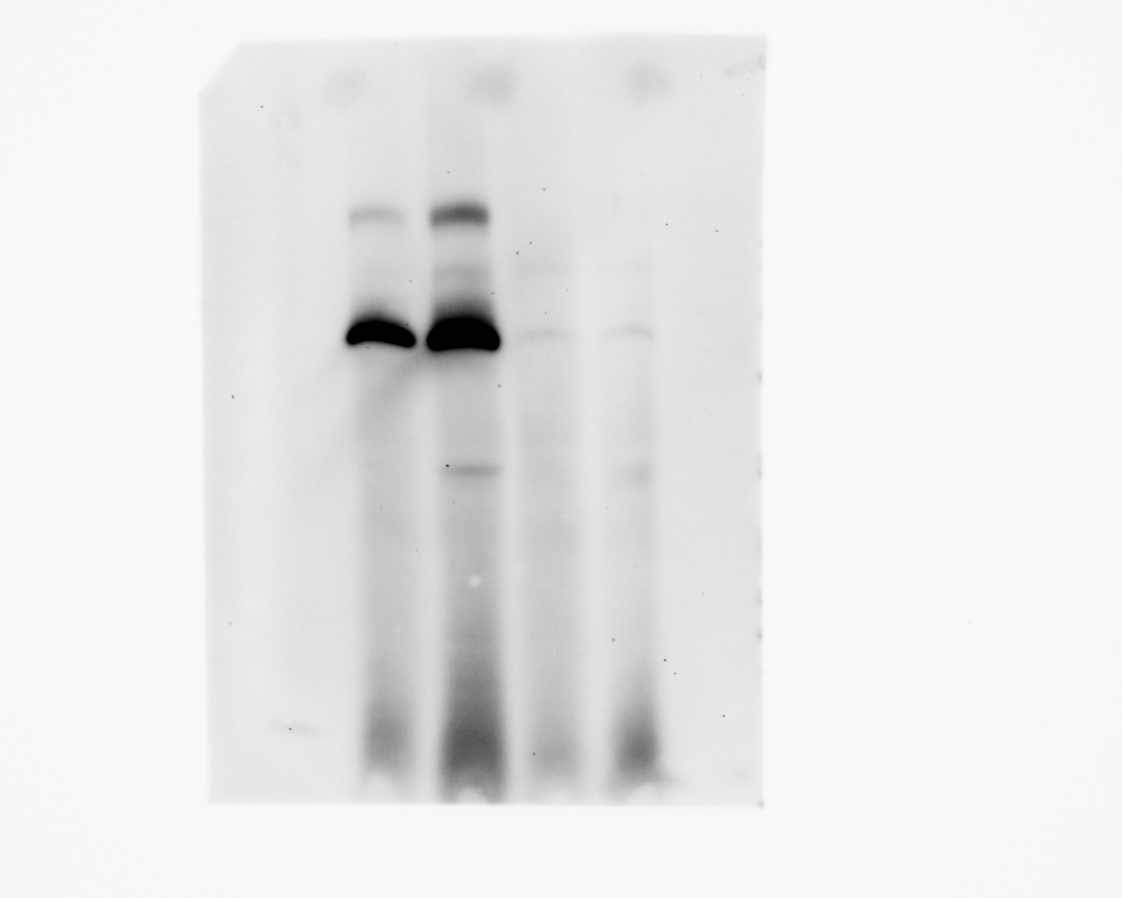

Supplement: Figure 2—figure supplement 1—source data 1. [file elife-84282-fig2-figsupp1-data1.zip › Figure 2-figure supplement 1-source data 1/raw data file5_FigS2Atp2_HAP4OE_BN-PAGE_4.27.23.jpg]

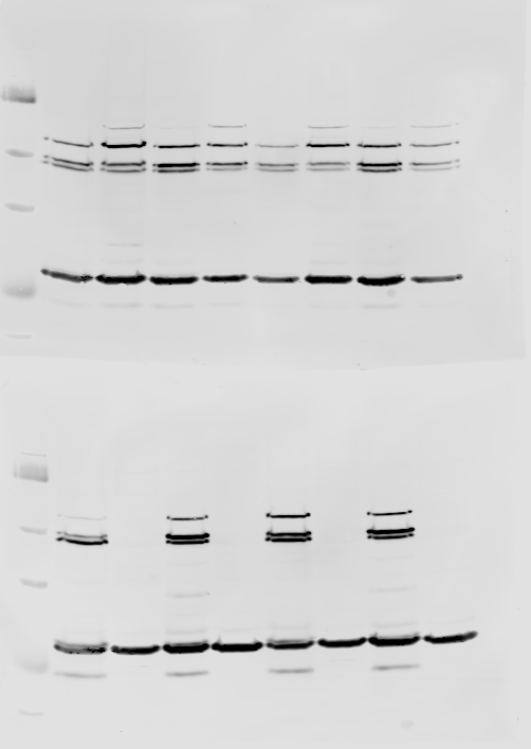

Supplement: Figure 2—figure supplement 1—source data 1. [file elife-84282-fig2-figsupp1-data1.zip › Figure 2-figure supplement 1-source data 1/raw data file3_FigS2_LA_Pi_wtrho0_sit4mct1_7.22.22.png]

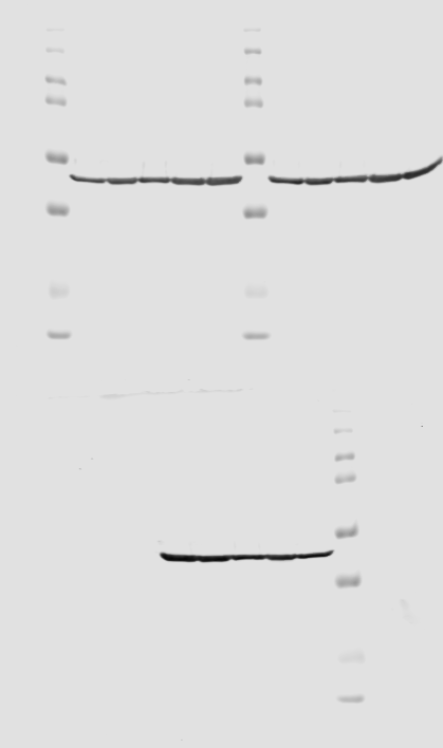

Supplement: Figure 3—source data 1. [file elife-84282-fig3-data1.zip › Figure 3-source data 1/raw data file2_Fig3_pgk1_ilv2-flag_Pi_triplicate_7.22.22.png]

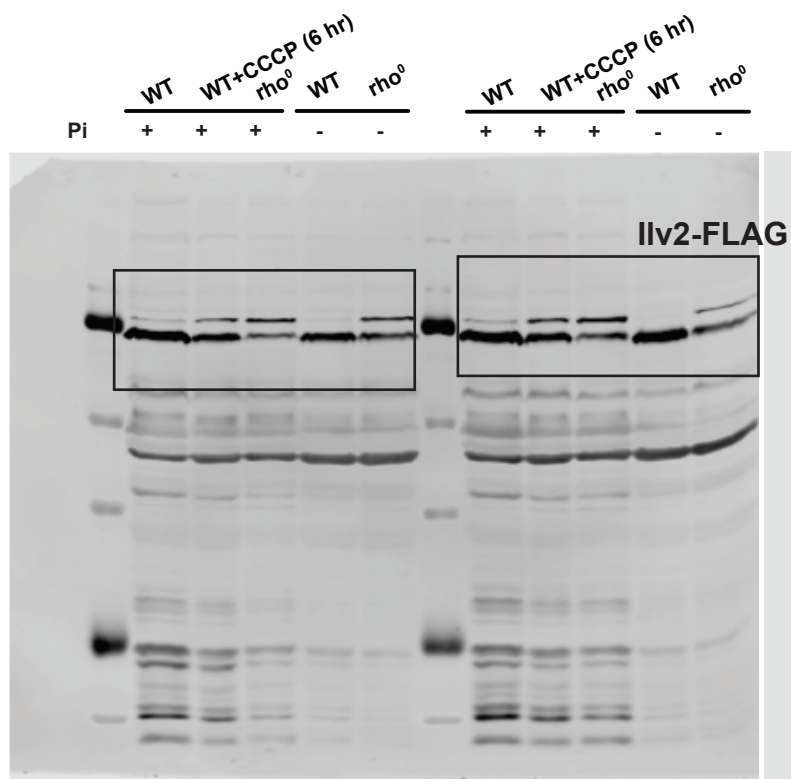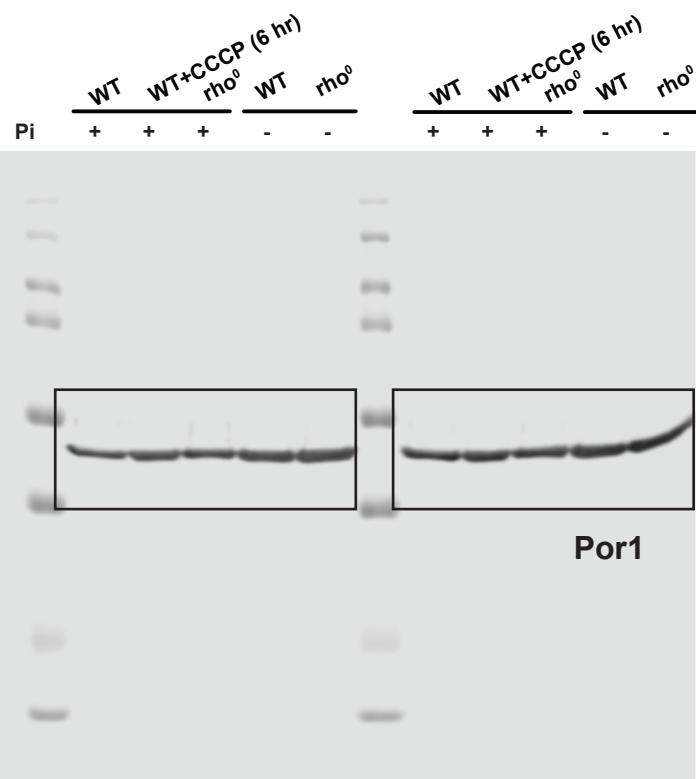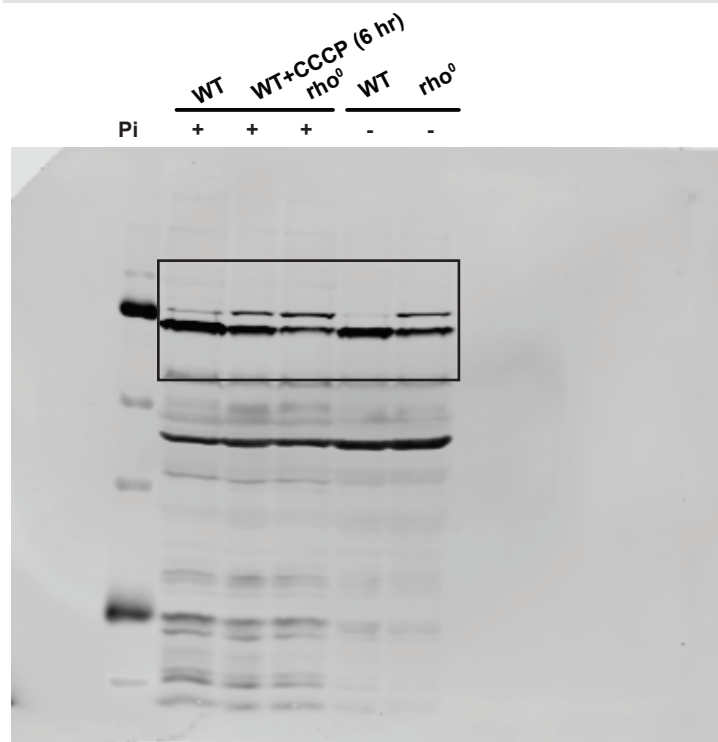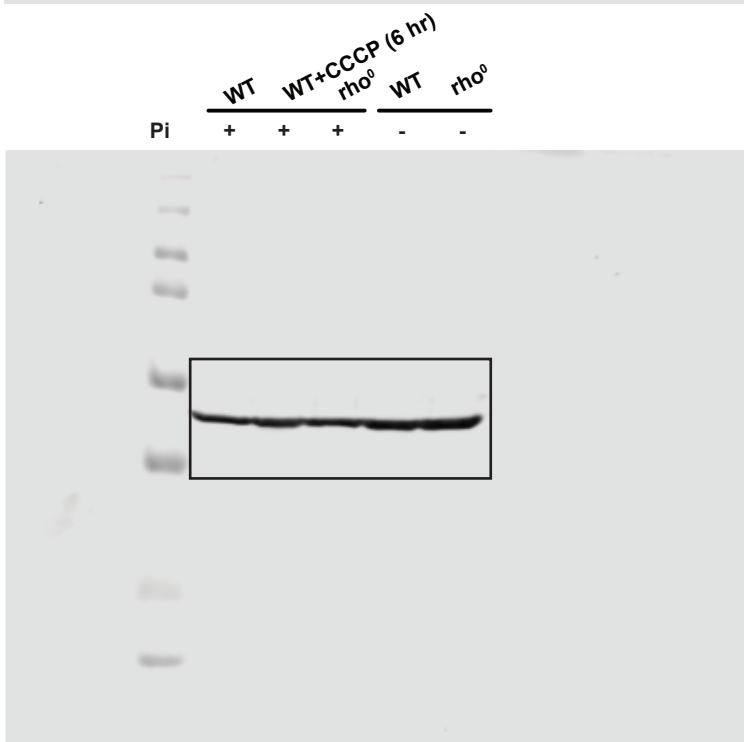

Supplement: Figure 3—source data 1. [file elife-84282-fig3-data1.zip › Figure 3-source data 1/raw data associated with Figure 3.pdf]

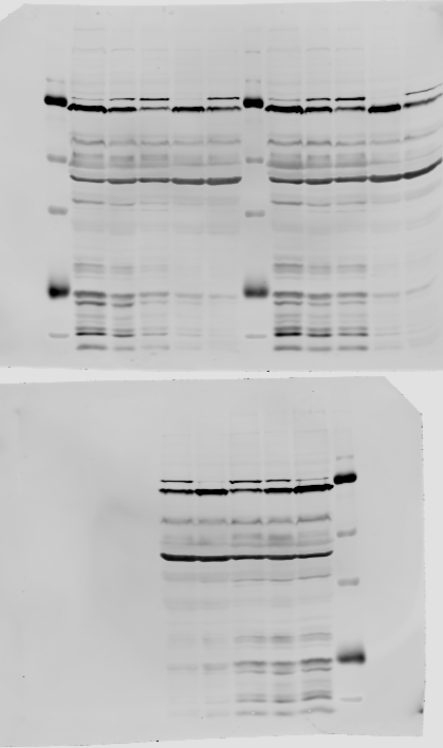

Supplement: Figure 3—source data 1. [file elife-84282-fig3-data1.zip › Figure 3-source data 1/raw data file1_Fig3_ilv2-flag_Pi_triplicate_7.22.22.png]

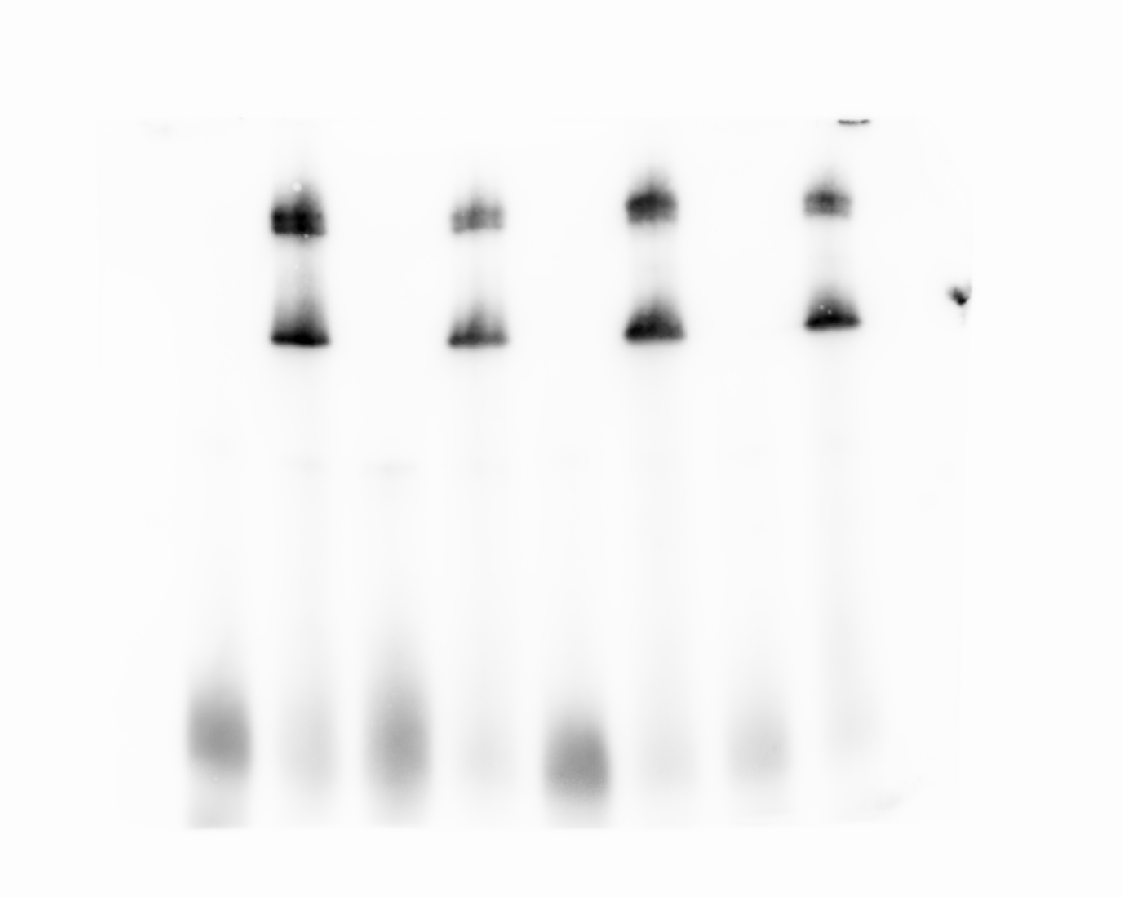

Supplement: Figure 4—source data 1. [file elife-84282-fig4-data1.zip › Figure 4-source data 1/raw data file1_Fig4_2022-07-21 190611 BNP-4(Chemiluminescence).tif]

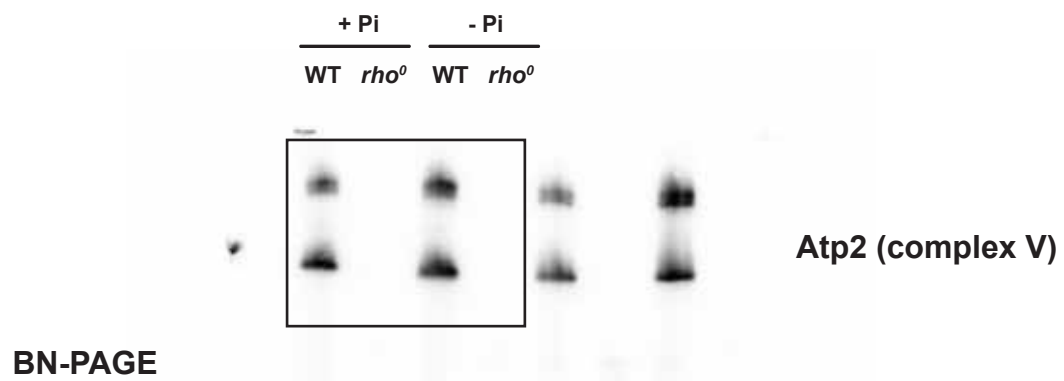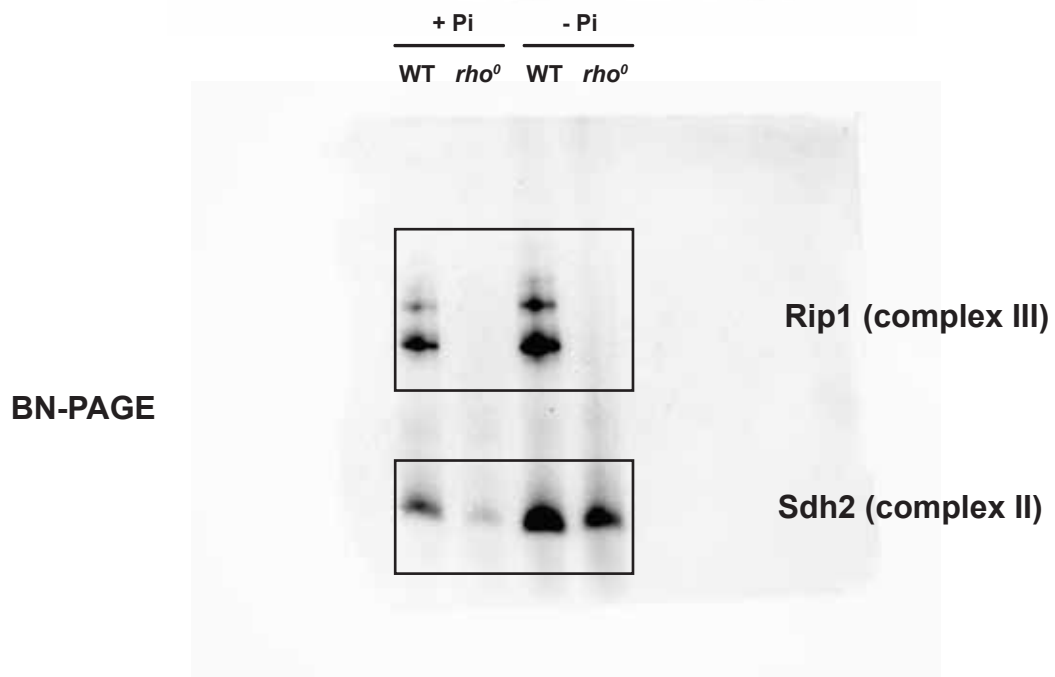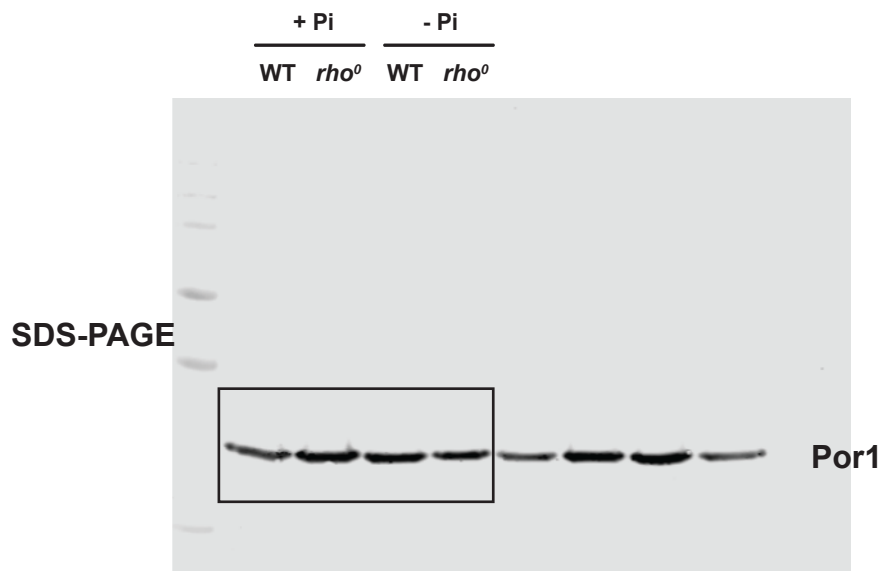

Supplement: Figure 4—source data 1. [file elife-84282-fig4-data1.zip › Figure 4-source data 1/raw data associated with Figure 4.pdf]

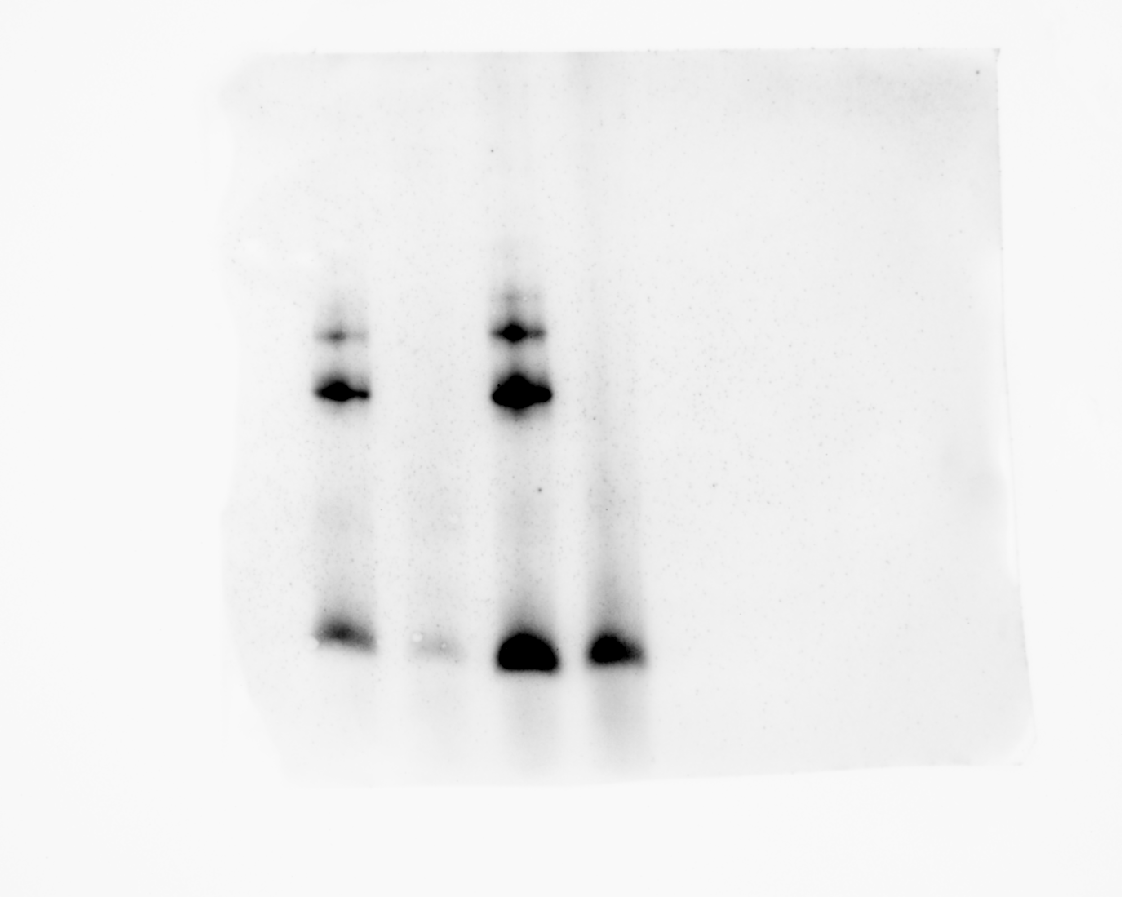

Supplement: Figure 4—source data 1. [file elife-84282-fig4-data1.zip › Figure 4-source data 1/raw data file2_Fig4_2022-07-21 190611 BNP-5(Chemiluminescence).tif]

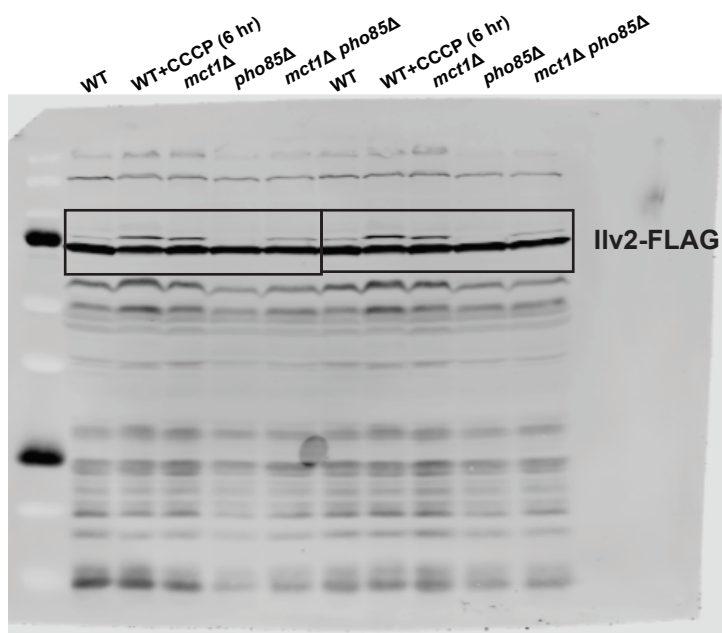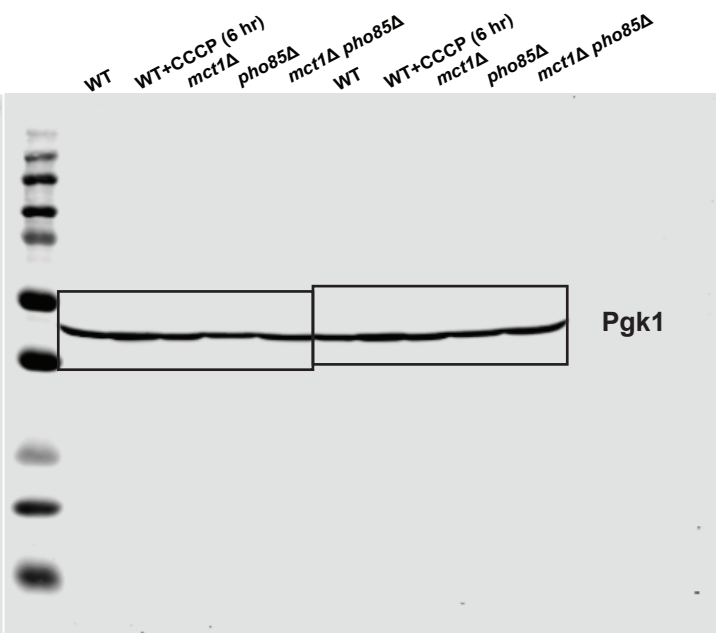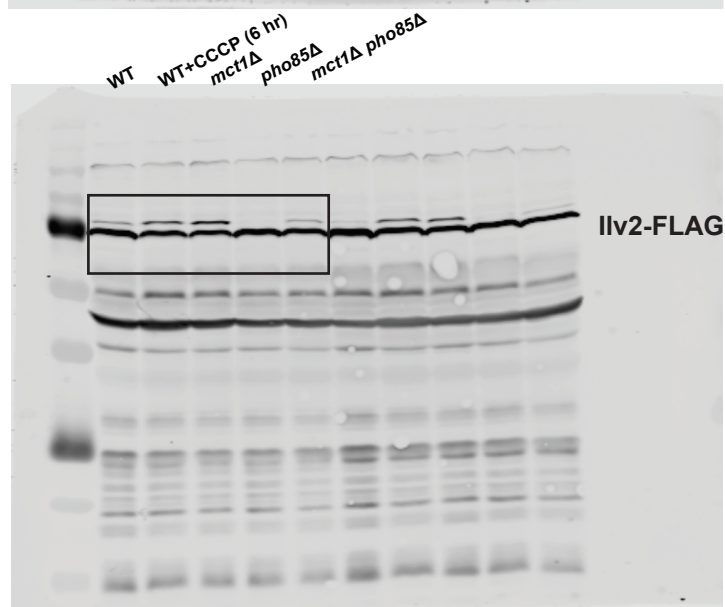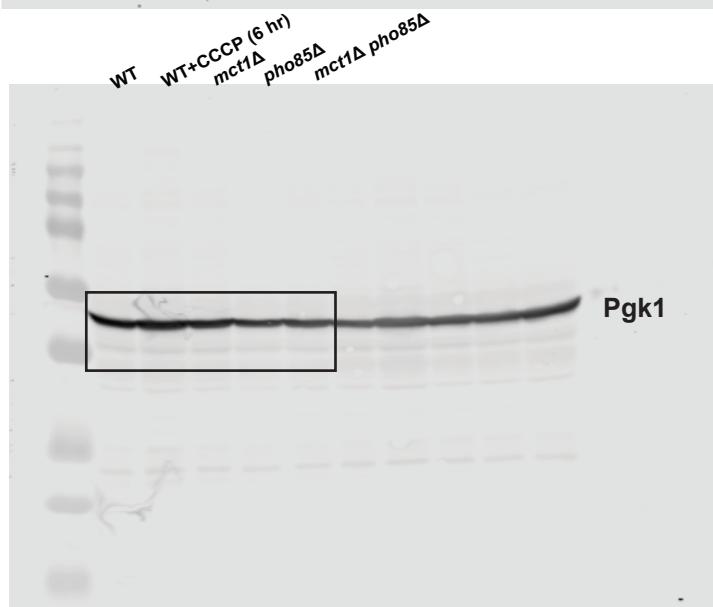

Supplement: Figure 5—source data 1. [file elife-84282-fig5-data1.zip › Figure 5-source data 1/raw data associated with Figure 5-1.pdf]

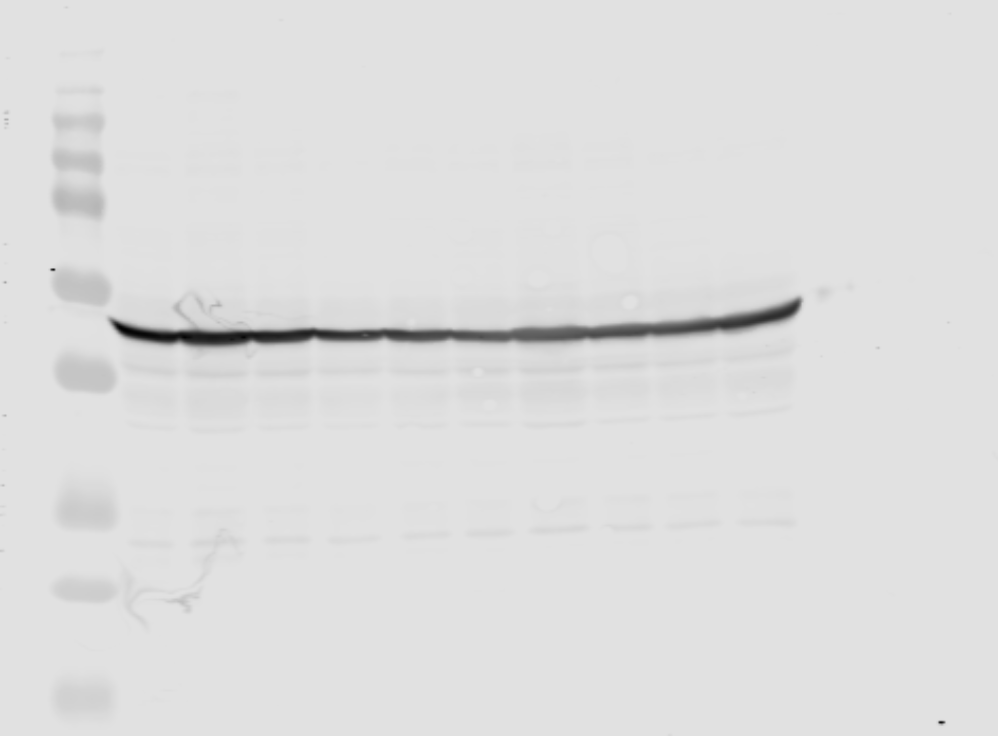

Supplement: Figure 5—source data 1. [file elife-84282-fig5-data1.zip › Figure 5-source data 1/raw data file4_Fig5_pgk1_ilv2-flag_pho85mct1_triplicate_3.23.22.png]

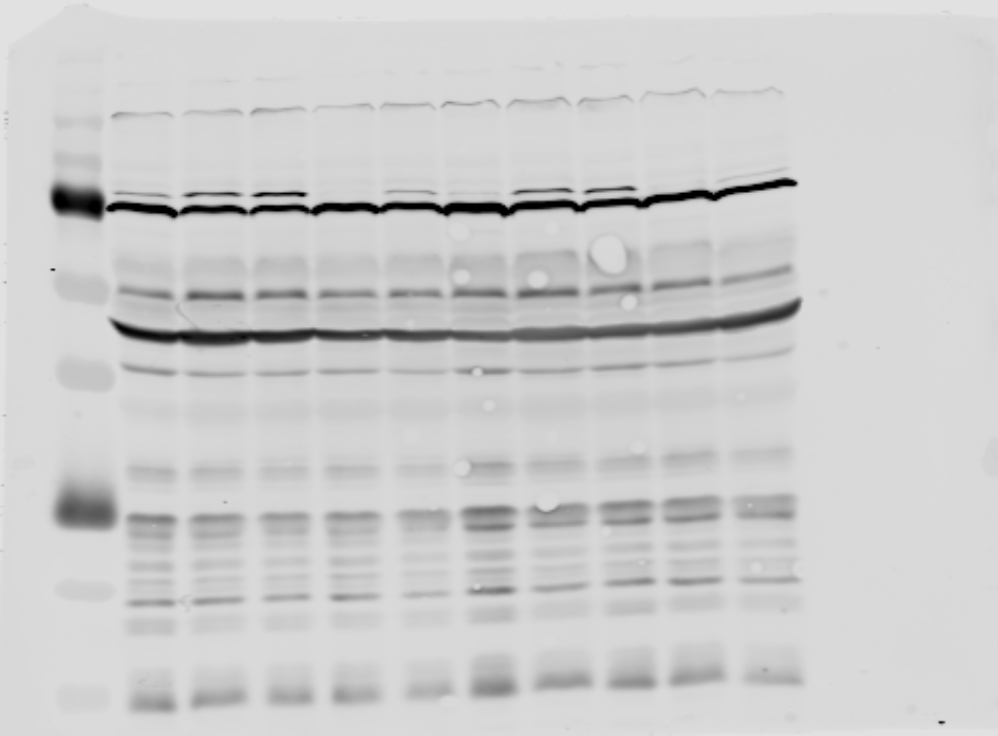

Supplement: Figure 5—source data 1. [file elife-84282-fig5-data1.zip › Figure 5-source data 1/raw data file3_Fig5_ilv2-flag_pho85mct1_triplicate_3.23.22.png]

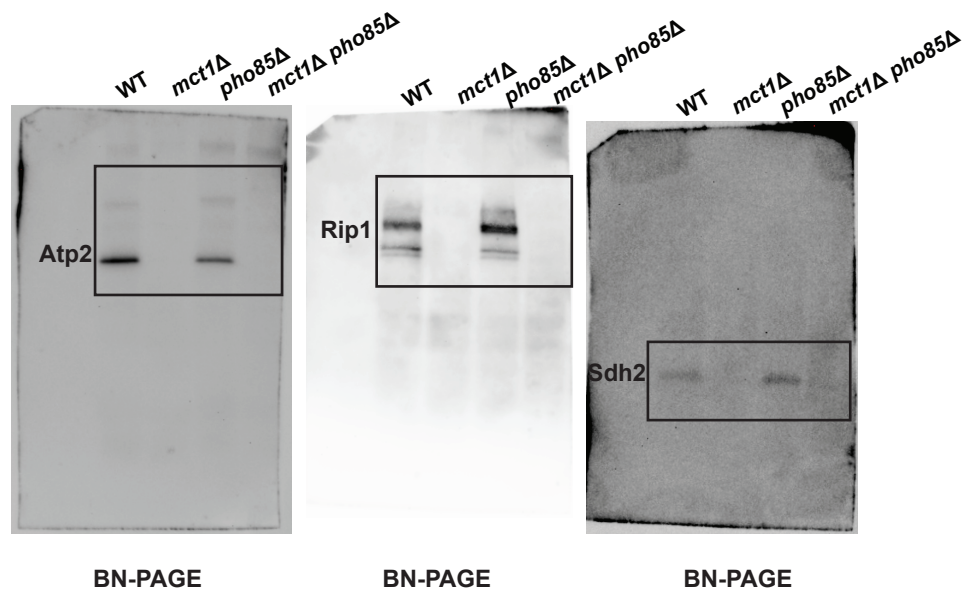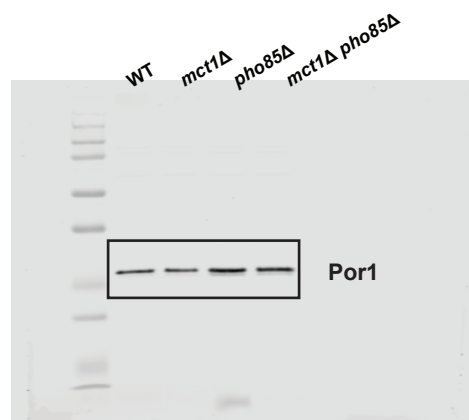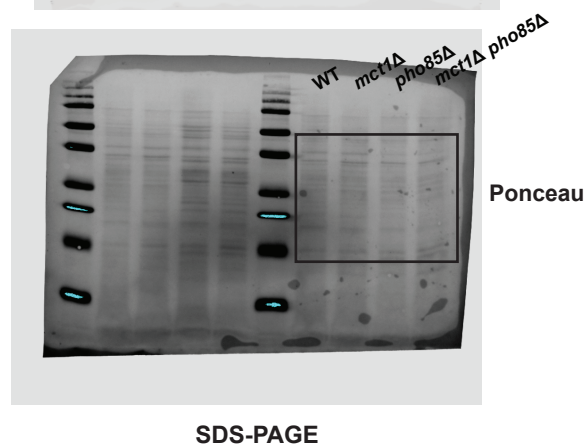

Supplement: Figure 5—source data 2. [file elife-84282-fig5-data2.zip › Figure 5-source data 2/raw data associated with Figure 5-2.pdf]

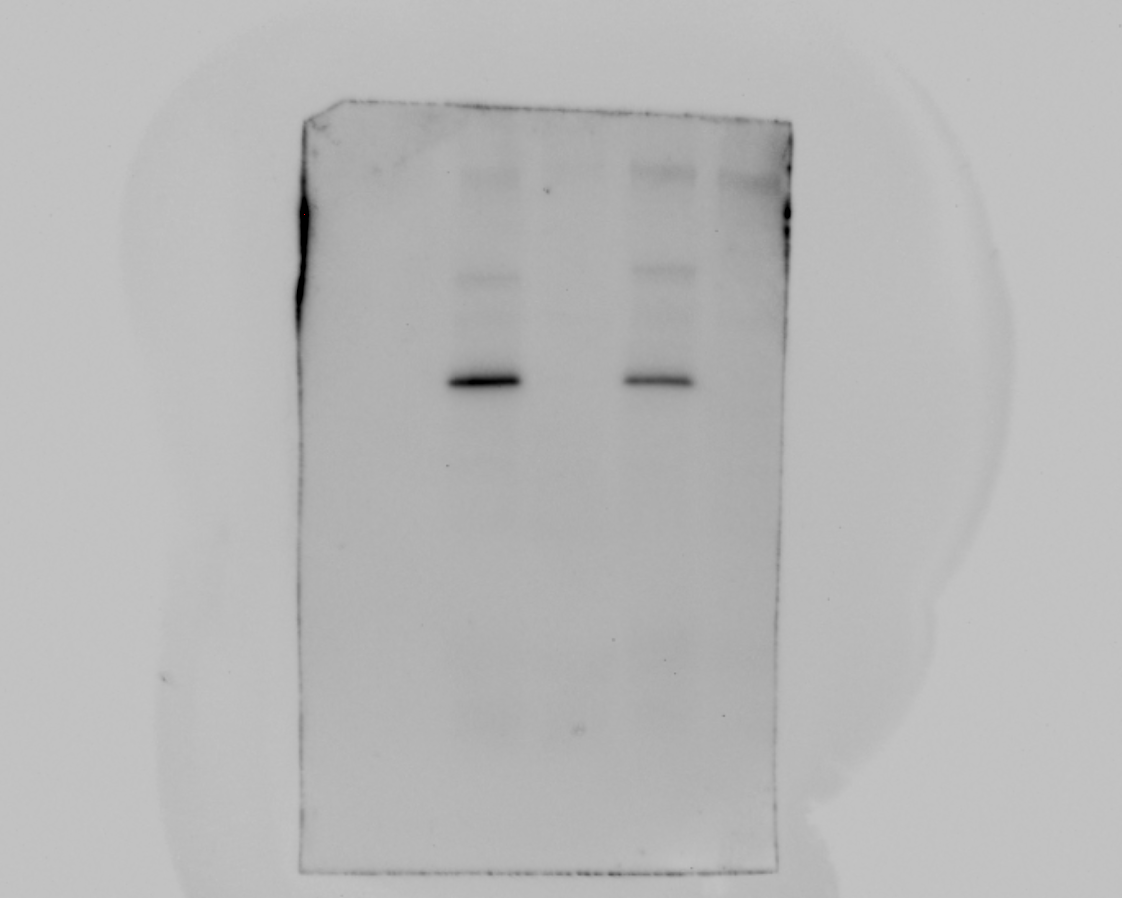

Supplement: Figure 5—source data 2. [file elife-84282-fig5-data2.zip › Figure 5-source data 2/raw data file1_Fig5_2021-04-15 190611 BNP(Chemiluminescence) (1).tif]

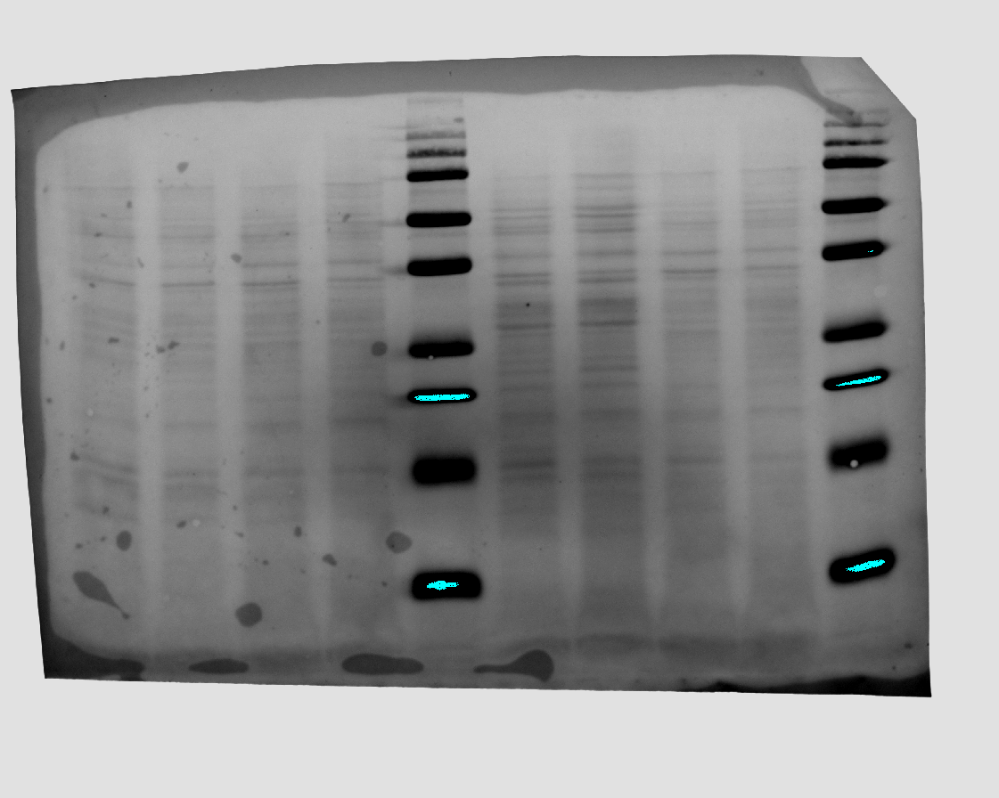

Supplement: Figure 5—source data 2. [file elife-84282-fig5-data2.zip › Figure 5-source data 2/raw data file5_Fig5_BN-PAGE_SDS-ponceau_sit4_pho85_7.19.22.png]

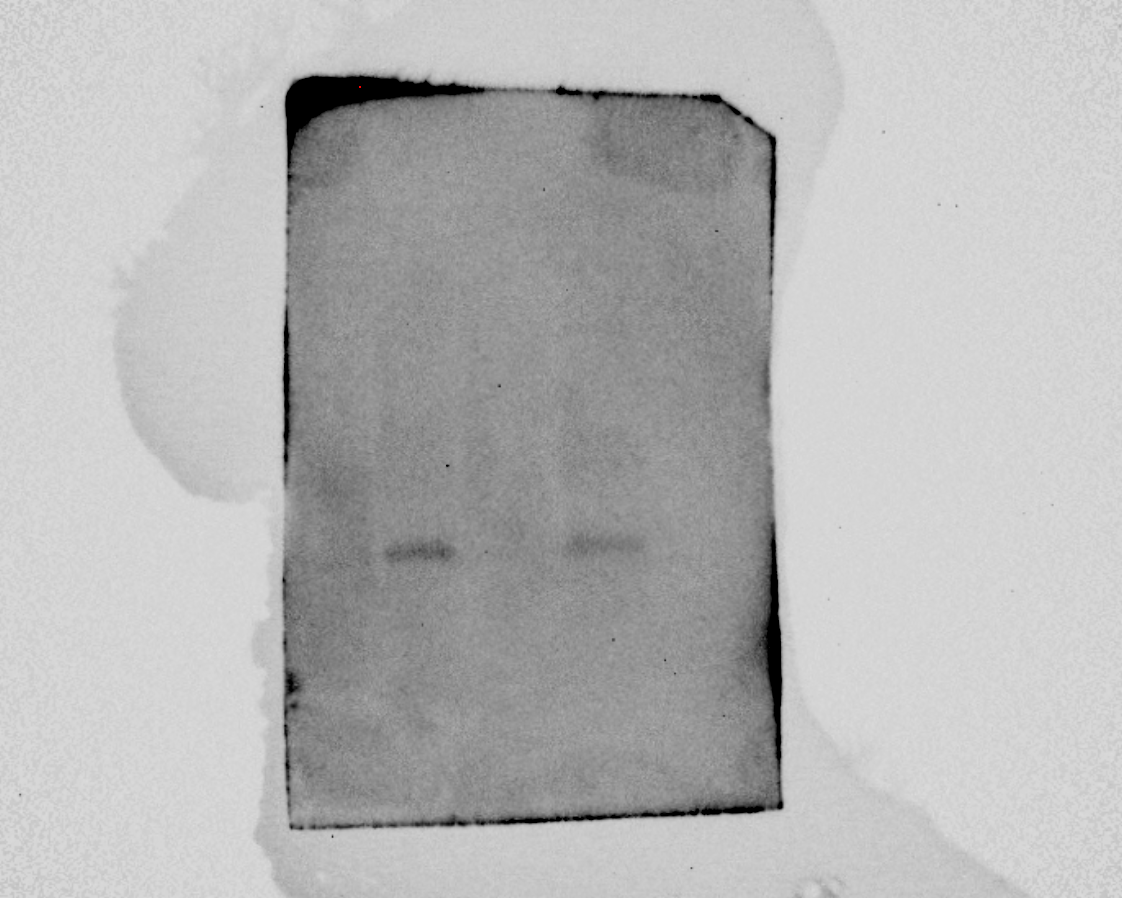

Supplement: Figure 5—source data 2. [file elife-84282-fig5-data2.zip › Figure 5-source data 2/raw data file3_Fig5_2021-04-19 190611 BNP(Chemiluminescence) (1).tif]

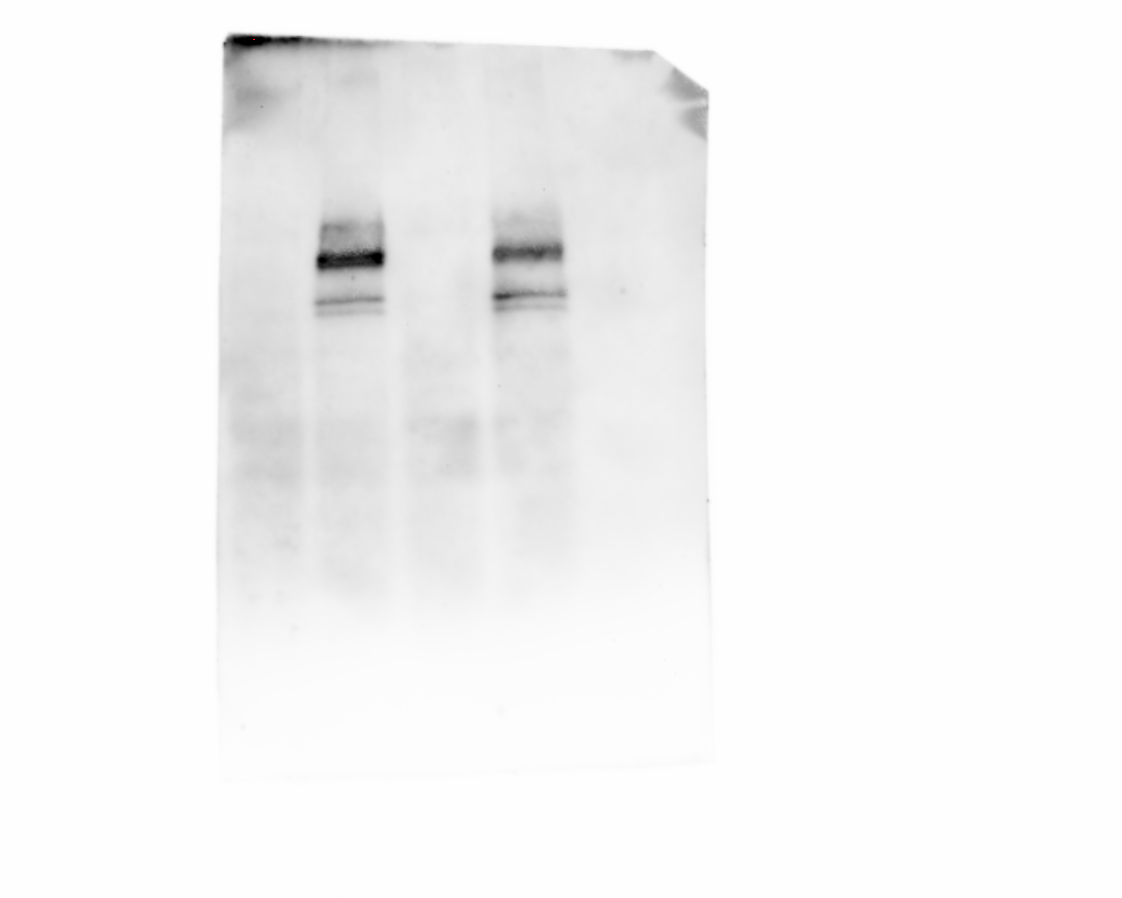

Supplement: Figure 5—source data 2. [file elife-84282-fig5-data2.zip › Figure 5-source data 2/raw data file2_Fig5_2021-04-15 190611 BNP-1(Chemiluminescence) (1).tif]

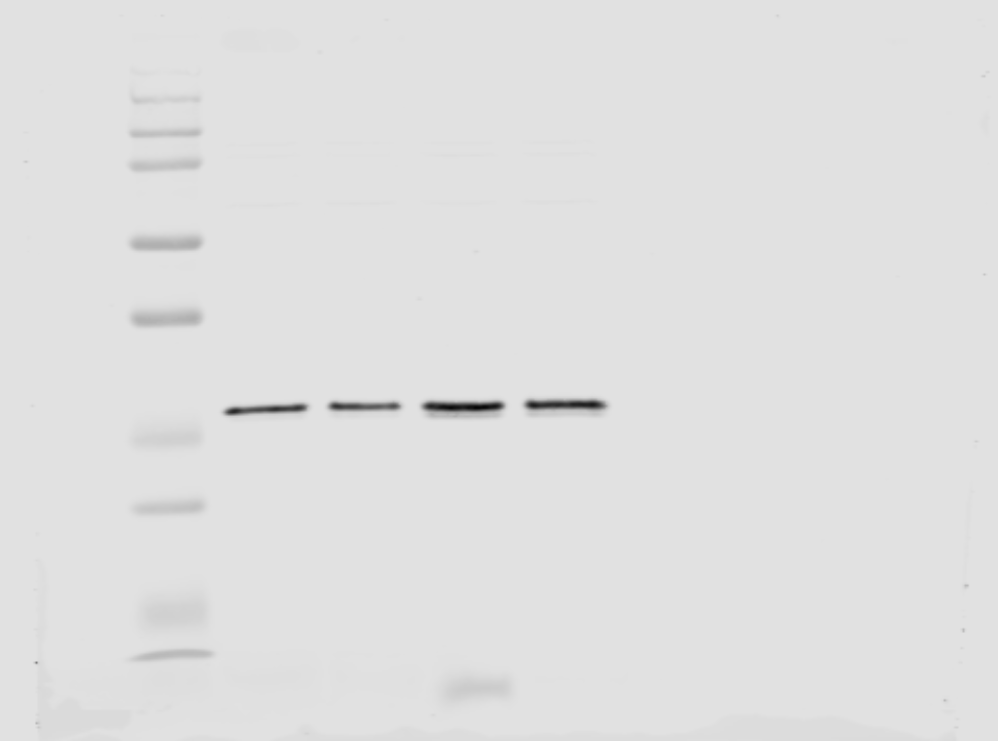

Supplement: Figure 5—source data 2. [file elife-84282-fig5-data2.zip › Figure 5-source data 2/raw data file4_Fig5_por1_ctrl_BNPAGE_4.15.21.png]
